# Assessment of Atmospheric and Surface Energy Budgets Using Observation-Based Data Products

**DOI:** 10.1007/s10712-024-09827-x

**Published:** 2024-04-17

**Authors:** Michael Mayer, Seiji Kato, Michael Bosilovich, Peter Bechtold, Johannes Mayer, Marc Schröder, Ali Behrangi, Martin Wild, Shinya Kobayashi, Zhujun Li, Tristan L’Ecuyer

**Affiliations:** 1 Research Department, European Centre for Medium-Range Weather Forecasts, Reading, RG2 9AX UK; 2 Department of Meteorology and Geophysics, University of Vienna, 1090 Vienna, Austria; 3 NASA Langley Research Center, Hampton, VA 23681-2199 USA; 4 NASA Global Modeling and Assimilation Office, Goddard Space Flight Center, Greenbelt, MD 20771 USA; 5 Satellite-Based Climate Monitoring, Deutscher Wetterdienst, 63067 Offenbach, Germany; 6 Department of Hydrology and Atmospheric Sciences, University of Arizona, Tucson, AZ 85721 USA; 7 Institute for Atmospheric and Climate Science, ETH Zurich, 8092 Zurich, Switzerland; 8 Numerical Prediction Development Center, Japan Meteorological Agency, Tsukuba City, 305-0052 Japan; 9 Department of Atmospheric and Oceanic Science, University of Wisconsin, Madison, WI 53706 USA

**Keywords:** Energy budget, Earth’s energy imbalance, Surface energy flux, Reanalysis, Remote sensing

## Abstract

Accurate diagnosis of regional atmospheric and surface energy budgets is critical for understanding the spatial distribution of heat uptake associated with the Earth’s energy imbalance (EEI). This contribution discusses frameworks and methods for consistent evaluation of key quantities of those budgets using observationally constrained data sets. It thereby touches upon assumptions made in data products which have implications for these evaluations. We evaluate 2001–2020 average regional total (TE) and dry static energy (DSE) budgets using satellite-based and reanalysis data. For the first time, a consistent framework is applied to the ensemble of the 5th generation European Reanalysis (ERA5), version 2 of modern-era retrospective analysis for research and applications (MERRA-2), and the Japanese 55-year Reanalysis (JRA55). Uncertainties of the computed budgets are assessed through inter-product spread and evaluation of physical constraints. Furthermore, we use the TE budget to infer fields of net surface energy flux. Results indicate biases < 1 W/m^2^ on the global, < 5 W/m^2^ on the continental, and ~ 15 W/m^2^ on the regional scale. Inferred net surface energy fluxes exhibit reduced large-scale biases compared to surface flux data based on remote sensing and models. We use the DSE budget to infer atmospheric diabatic heating from condensational processes. Comparison to observation-based precipitation data indicates larger uncertainties (10–15 Wm^−2^ globally) in the DSE budget compared to the TE budget, which is reflected by increased spread in reanalysis-based fields. Continued validation efforts of atmospheric energy budgets are needed to document progress in new and upcoming observational products, and to understand their limitations when performing EEI research.

**Article Highlights**

- Consistent frameworks to evaluate atmospheric energy budgets from observationally constrained data sets are presented
- Total and dry static energy budgets are used to infer net surface energy flux and diabatic heating from precipitation, respectively
- Uncertainties of inferred net surface energy flux are demonstrably lower than those based on remote sensing products

## Introduction

The atmosphere redistributes petawatts of energy globally, with annual mean zonally integrated poleward transports peaking over 4 petawatts in both hemispheres (e.g., Mayer et al. 2021). It thereby accomplishes a large fraction of planetary heat transport from the tropics to the poles (e.g., Trenberth et al. 2019), as required by latitudinal changes in net energy input at the top-of-atmosphere (TOA) associated with Earth’s spherical geometry (Peixoto and Oort 1992). Furthermore, regional patterns of the divergence of atmospheric total energy transports are related to net surface energy fluxes into and out of the ocean: Climatological convergence (divergence) tends to occur over regions of enhanced ocean heat uptake (release), such as the equatorial cold tongues (Western Boundary Currents) (Trenberth and Stepaniak 2003). In the context of Earth’s energy imbalance (EEI), divergent atmospheric energy transports determine how spatial variations and trends of net radiation at the TOA are redistributed, and thereby how heat input at the ocean surface is modulated. The atmospheric divergence is thus a critical quantity for understanding regional patterns of ocean heat uptake. Indeed, decadal changes in atmospheric circulation and/or gradients lead to pronounced decadal variations in divergent atmospheric energy transport and largely determine spatial patterns of decadal trends in net surface energy flux (Loeb et al. 2022).

As opposed to TOA, where high-quality observations exist, most notably products from the Clouds and the Earth's Radiant Energy System (CERES) program (Loeb et al. 2018), there do not exist in situ observations of atmospheric energy transports on a global scale, and direct measurements of air–sea fluxes are scarce. Atmospheric energy transports are typically diagnosed from reanalyses, which comes with several caveats regarding methods and data (Trenberth 1991; Mayer et al. 2017; Trenberth and Fasullo 2018; Kato et al. 2021). In the absence of direct measurements, e.g., through eddy covariance methods (which themselves exhibit large uncertainties, see Mauder et al. 2020 for a recent review), air–sea fluxes can be estimated using bulk formulae, using meteorological input data based on models or remotely sensed data, but such estimates exhibit considerable uncertainties, leading to large global imbalances of the obtained fluxes in this way (Yu 2019). An alternative approach infers net surface energy flux from the atmospheric energy budget, which has some advantages compared to the “direct” approach and has been followed in numerous studies (e.g., Trenberth and Fasullo 2017; Cheng et al. 2019; Mayer et al. 2022).

The focus of this paper is twofold. We first discuss in detail the practical evaluation of atmospheric total and dry static energy budgets from observationally constrained products. For this, we begin from a complete formulation of the total atmospheric energy budget, derivation of which has been presented elsewhere (e.g., Bannon 2002; Lauritzen et al. 2018; Kato et al 2021; Lauritzen et al. 2022). We then discuss simplifications typically made in models underlying reanalysis products to provide a framework for practical evaluation of atmospheric energy budgets and discuss pragmatic choices made in the data products and the diagnostic framework. This includes discussion of technical aspects such as mass corrections. The formulation of diagnostic atmospheric energy budgets has received some attention in recent years, i.e., because data quality has become sufficient to reveal biases in previously employed frameworks (Mayer et al. 2017). In this context, we reconcile apparent discrepancies between budget formulations presented in recent works (Mayer et al. 2017; Trenberth and Fasullo 2018; Kato et al. 2021). The second aim of this paper is to provide an overview of uncertainty of the diagnosed quantities and flux estimates using state-of-the-art data products. To this end, we rely on (i) inter-comparison of data products, (ii) assessment of physical constraints, and (iii) employing the budget to infer a quantity which can be compared against an observational product (e.g., comparison of diabatic heating from precipitation against an observational precipitation product).

Section 2 provides an overview of employed data products and considerations regarding the presented diagnostics. Section 3 discusses atmospheric budgets, including the atmospheric budget and its relevance for energy budget diagnostics (3.1), the total energy budget (3.2), and the dry static energy budget (3.3). Section 4 covers the surface energy budget, including an assessment of physical constraints of the total energy budget. Conclusions, recommendations, and an outlook are provided in Sect. 5.

## Data, Study Period, Diagnostics

We employ a vertically integrated framework to diagnose atmospheric budgets. The vertical coordinate is thus eliminated. Energy fluxes discussed in this study are, therefore, radiative flux at the TOA, horizontal transports and storage within the atmosphere, and fluxes at the surface.

We use TOA fluxes from the CERES Energy-Balanced and Filled product in Edition 4.2 (CERES-EBAF-TOA; Loeb et al. 2018). They are tuned (with a one-time global adjustment) to match the global net TOA flux averaged from July 2005 through June 2015 with an observational estimate of the sum of the rate of global ocean heating, ice heating and melt, and atmospheric and lithospheric heating averaged over this period.

Transports within the atmosphere (and their divergence) of total energy (precisely: moist static plus kinetic) and dry static energy as well as storage rates of total and dry static energy are obtained from three atmospheric reanalyses ERA5 (Hersbach et al. 2020), MERRA-2 (Gelaro et al. 2017), and JRA55 (Kobayashi et al. 2015). They differ in many aspects, including data assimilation system, resolution, boundary conditions, and treatment of observations. Please refer to the corresponding references for details. The three products are of different vintage, with ERA5 being the most recent of the group, but successors of the others are underway or currently being produced (e.g., JRA-3Q; Kosaka et al. 2024). The required quantities (winds, pressure, and thermodynamic fields) rely on analyzed state quantities from reanalyses, i.e., they are strongly constrained by observations. The evaluation of ERA5 and JRA55 data is performed using data on the model native vertical and horizontal grid at maximum available temporal resolution (1-hourly and 6-hourly, respectively) to avoid interpolation errors. The divergence fields are mass-adjusted as described in Sect. 3.2.4. Native grid 3D fields at high temporal resolution that are required for the presented budget diagnostics were not available from MERRA-2. Yet, the MERRA-2 archive provides unadjusted vertically integrated monthly mean divergences, which are computed on the native grid at each model time step and temporally accumulated (Global Modeling and Assimilation Office (GMAO) 2015a). Methods for mass adjustments are discussed in Sect. 3.2.4.

Budgets derived from some reanalyses suffer from spectral noise of the divergence term, and typically useful resolution is limited to around 2.5° (e.g., Trenberth and Stepaniak 2003). However, progress has been made in recent years to reduce spectral noise in budgets derived from ERA5, but this requires advanced numerical methods (see Mayer et al. 2021 for computational details), allowing for an increase of useful resolution to about 1°. MERRA-2 budgets do not suffer from these problems thanks to the finite volume dynamical core of the underlying model and computation of the divergence at each model time step (see the above paragraph), and in principle, no truncation of the MERRA-2 results is necessary.

Diabatic heating from condensation processes is a crucial element of the dry static energy budget. In this context, we use the latest version (V3.2) of the Global Precipitation Climatology Project (GPCP) product (Huffman et al. 2023).

Surface energy fluxes are obtained from different types of data sets. We use satellite-based data sets of net surface radiation, namely CERES-EBAF surface in Edition 4.2 (CERES-EBAF-sfc; Kato et al. 2018) and CM SAF cLoud, Albedo and surface radiation data set from AVHRR data in version 3 (CLARA-A3; Karlsson et al. 2023), and turbulent fluxes derived from remote sensing data, namely OAflux version 3 (Yu and Weller 2007), Japanese Ocean Flux Data Sets with Use of Remote-Sensing Observations 3 (J-OFURO3) in version 1.1 (Tomita et al. 2019), IFREMER v4.1 (Bentamy et al. 2013), and SeaFlux v3 (Curry et al. 2004). CERES-EBAF-sfc fluxes are constrained by observed TOA fluxes by the method described in Kato et al. (2018).

Reanalyses also provide surface energy fluxes as output from short-term (typically 12-hourly) forecasts of the underlying models. By nature, these fields are less strongly constrained by observations (but through the initial conditions of the forecasts). We employ model-based fluxes from a range of reanalyses with varying degrees of observational data ingestion (see Table 1 for a complete list).

**Table 1:** Overview of employed data products, their type, reference papers, and the evaluated budget terms

Product	Type	References	Evaluated terms
CERES-EBAF-TOA ed4.2	Satellite, with global mean adjustment	Loeb et al. (2018)	Rad_TOA_
ERA5	Reanalysis	Hersbach et al. (2020)	$\frac{\partial }{\partial t}{\text{AE}}$, TEDIV, DSEDIV, LH, SH, Rad_S_, *P*_snow_
JRA55	Reanalysis	Kobayashi et al. (2015)	$\frac{\partial }{\partial t}{\text{AE}}$, TEDIV, DSEDIV, LH, SH, Rad_S_, *P*_snow_
MERRA-2	Reanalysis	Gelaro et al. (2017), Global Modeling and Assimilation Office (GMAO) (2015a, b, c)	$\frac{\partial }{\partial t}AE$, TEDIV, DSEDIV, LH, SH, Rad_S_, *P*_snow_
GPCP V3.2	Satellite plus rain gauge	Huffman et al. (2023)	*P*
IFREMER	Satellite	Bentamy et al. (2013)	LH, SH
OAflux	Satellite	Yu and Weller (2007)	LH, SH
J-OFURO	Satellite	Tomita et al. (2019)	LH, SH
SeaFlux	Satellite	Curry et al. (2004)	LH, SH
CERES-EBAF-sfc ed4.2	Satellite plus radiative transfer model	Kato et al. (2018)	Rad_S_
CLARA-A3	Satellite plus radiative transfer model	Karlsson et al. (2023)	Rad_S_
MERRA	Reanalysis	Rienecker et al. (2011)	LH, SH, Rad_S_, *P*_snow_
MERRA-2-AMIP	Reanalysis (no data assimilation)	Collow et al. (2017)	LH, SH, Rad_S_, *P*_snow_
NCEP R2	Reanalysis	Kanamitsu et al. (2002)	LH, SH, Rad_S_, *P*_snow_
20CRv3	Reanalysis (surface data only)	Slivinski et al. (2019)	LH, SH, Rad_S_, *P*_snow_
ERA20C	Reanalysis (surface data only)	Poli et al. (2016)	LH, SH, Rad_S_, *P*_snow_
ERA20CM	Reanalysis (no data assimilation)	Hersbach et al. (2015)	LH, SH, Rad_S_, *P*_snow_

An alternative to satellite products and fluxes from model forecasts is to infer the net surface energy flux from the atmospheric energy budget (storage rates and divergences based on analyses) in combination with observed TOA fluxes, which will be elaborated on in Sect. 4. Long-term means of relevant budget terms are averaged over the standard period 2001–2020. This is the twenty-year period starting with the first full year of CERES data. This also coincides with a time when atmospheric reanalyses are relatively stable temporally. Some of the employed satellite products cover a shorter period, which dictates the averaging period for the respective diagnostics.

Given the comprehensive discussion of methods, we limit diagnostics to multi-year average fields (2001–2020 averages wherever data availability allows). While computations such as divergences are performed on the respective native grids (see above), all results and data products are interpolated to a common 1 × 1° horizontal grid to facilitate inter-comparison. We consider long-term mean global averages of fields to check physical constraints. Unlike reanalyses, some of the employed satellite products have no full global coverage. For example, the satellite-based turbulent flux data have no data over land and in sea ice covered regions. For consistent inter-comparisons of these products with other estimates, we mask out grid points, where any of the involved products does not contain at least one valid value for each calendar month. Moreover, unbiased long-term means at grid points with temporally varying data coverage are ensured by first computing a mean annual cycle (using the available data for each calendar month) and subsequently long-term means as an average of the monthly values. This makes sure that no calendar month is over- or underrepresented. The same spatiotemporal mask is applied to all products used for the respective diagnostics.

## Atmospheric Energy Budgets

### Atmospheric Mass Budget

We begin with a short discussion of the atmospheric moisture and mass budget and its relevance for the energy budget. The mass budget of the atmosphere including water in all states reads as follows:

1 $$P+E+\frac{1}{g}\frac{\partial }{\partial t}{p}_{{\text{s}}}=-\nabla \cdot \frac{1}{g}{\int }_{0}^{{p}_{{\text{s}}}}\overset{\lower0.5em\hbox{$\smash{\scriptscriptstyle\rightharpoonup}$}}{{\mathbf{v}}}{\text{d}}p$$

Precipitation *P* and evaporation *E* represent surface fluxes of all species of water and are defined positive downward. Surface pressure *p*_s_ is proportional to the mass of the moist air column, scaled with gravitational acceleration *g*. The horizontal wind $$\overset{\lower0.5em\hbox{$\smash{\scriptscriptstyle\rightharpoonup}$}}{{\mathbf{v}}}$$ denotes barycentric velocity and represents the horizontal mass flux of moist air (including dry air and all species of water). Further degrees of freedom can be introduced by allowing for different velocities of dry air and water species (Kato et al. 2021). However, when the mass-weighted moist air velocity is considered it can be assumed to be the velocity of dry air because the mixing ratio is of the order of or less than 10^–2^ (Bannon 2002). In addition, observational products and reanalyses typically do not provide velocity of hydrometeors. The model and assimilation system underlying MERRA-2, satisfies Eq. (1) and thus conserves atmospheric dry mass (Takacs et al. 2016, see their Fig. 5).

An important approximation in the Integrated Forecasting System (IFS), the model underlying ERA5, is that surface precipitation and evaporation do not change the total mass of the atmosphere as a change in water mass is implicitly replaced by an equivalent change in dry air mass (Malardel et al. 2019). As a result, lateral convergence and divergence of moisture is balanced by unphysical divergence and convergence of dry air, respectively, instead of *P* + *E*. Consequently, surface pressure does not vary in response to moisture changes in the above column. The moist continuity equation in the IFS hence reads as follows (ECMWF 2021a):

2 $$\frac{1}{g}\frac{\partial }{\partial t}{p}_{{\text{s}},{\text{IFS}}}=-\nabla \cdot \frac{1}{g}{\int }_{0}^{{p}_{{\text{s}}}}\overset{\lower0.5em\hbox{$\smash{\scriptscriptstyle\rightharpoonup}$}}{{\mathbf{v}}}{\text{d}}p$$

The divergence term in Eq. (2) thus contains contributions from physical dry air divergence (which can change surface pressure) but no contributions from moisture flux divergence. Hence, the long-term mean of lateral mass flux divergence from ERA5 does not show the signature of *P* + *E* (Mayer et al. 2021; their Fig. 2a), as one would expect from Eq. (1) and physical conception. The JMA spectral model makes similar approximations as the IFS and hence satisfies Eq. (2) as well (JMA 2007). This has implications for mass corrections typically applied for energy budget diagnostics, which will be discussed in Sect. 3.2.4.

### Atmospheric Total Energy Budget

#### Complete Formulation

We use the total atmospheric energy budget equation using liquid water at 0 °C as a reference state from Lauritzen et al. (2022; their Eq. 12) as a starting point, but write it in vertical pressure coordinates, use specific moisture quantities instead of mixing ratios, and include lateral transports (i.e., a local instead of a globally integrated formulation):

3 $$\begin{aligned} & {\text{Rad}}_{{{\text{TOA}}}} - {\text{Rad}}_{{\text{S}}} - {\text{SH}} - {\text{LH}} - L_{{\text{f}}} \left( {T_{{\text{p}}} } \right)P_{{{\text{snow}}}} - P\left[ {h_{00} + c_{{\text{l}}} (T_{{\text{p}}} - T_{00} } \right) + \phi_{{\text{s}}} + k_{{\text{s}}} \left] { - E} \right[h_{00} + c_{{\text{l}}} (T_{{\text{s}}} - T_{00} ) + \phi_{{\text{s}}} + k_{{\text{s}}} ] \\ & \quad = \frac{\partial }{\partial t}\frac{1}{g}\mathop \smallint \limits_{0}^{{p_{s} }} \left( {\left( {1 - q_{{\text{v}}} - q_{{\text{l}}} - q_{{\text{f}}} } \right)c_{{\text{a}}} (T_{{\text{a}}} - T_{00} ) + \left( {q_{{\text{v}}} + q_{{\text{l}}} + q_{{\text{f}}} } \right)c_{{\text{l}}} (T_{{\text{a}}} - T_{00} ) + L_{{\text{v}}} \left( {T_{{\text{a}}} } \right)q_{{\text{v}}} + L_{{\text{f}}} \left( {T_{{\text{a}}} } \right)q_{{\text{f}}} + \left( {q_{{\text{v}}} + q_{{\text{l}}} + q_{{\text{f}}} } \right)h_{00} + \phi_{{\text{s}}} + k} \right)\mathop{v}\limits^{\rightharpoonup} {\text{d}}p \\ & \quad + \nabla \cdot \frac{1}{g}\mathop \smallint \limits_{0}^{{p_{s} }} \left( {\left( {1 - q_{{\text{v}}} - q_{{\text{l}}} - q_{{\text{f}}} } \right)c_{{\text{a}}} (T_{a} - T_{00} ) + \left( {q_{{\text{v}}} + q_{{\text{l}}} + q_{{\text{f}}} } \right)c_{{\text{l}}} (T_{{\text{a}}} - T_{00} ) + L_{{\text{v}}} \left( {T_{{\text{a}}} } \right)q_{{\text{v}}} + L_{{\text{f}}} \left( {T_{{\text{a}}} } \right)q_{{\text{f}}} + \left( {q_{{\text{v}}} + q_{{\text{l}}} + q_{{\text{f}}} } \right)h_{00} + \phi + k} \right)\mathop{v}\limits^{\rightharpoonup} {\text{d}}p \\ \end{aligned}$$

The left-hand side (lhs) of Eq. (3) contains the vertical fluxes: net TOA radiative flux ${{\text{Rad}}}_{{\text{TOA}}}$, net surface radiative flux ${{\text{Rad}}}_{{\text{S}}}$, sensible heat flux SH, latent heat flux LH (computed as *L*_v_(*T*_00_)*E*, with *T*_00_ being the reference temperature), and latent heat flux associated with snowfall ${P}_{{\text{snow}}}$. The lhs also contains non-latent contributions of *P* (*F*_P_) and *E* (*F*_E_) to the energy budget, which include kinetic energy, surface geopotential ${\upphi }_{{\text{s}}}$, and enthalpy. Evaporation occurs at skin temperature *T*_s_, and for temperature of precipitation (*T*_P_) near-surface wet bulb temperature is deemed a good approximation (Gosnell et al. 1995). Figure 6 of Mayer et al. (2017) shows *F*_P_ and Fig. 3 of Kato et al. (2021) shows regional net enthalpy flux associated water mass exchanges at the surface, i.e., ${F}_{{\text{E}}}+{F}_{{\text{P}}}$. However, we will not evaluate these fluxes here as they are typically not provided by models or reanalyses and there are ambiguities related to the choice of reference temperature due to typically nonzero mass flux associated with *P* and *E*, which is reflected by the reference enthalpy term ${h}_{00}$ (discussed below). However, as will be discussed later, *F*_E_ and *F*_P_ are real physical terms, and their energetic effect on other budget terms may be implicitly included in observational products.

The right-hand side (rhs) contains storage rate of total atmospheric energy ($\frac{\partial }{\partial {\text{t}}}{\text{AE}}$) and divergence of moist static plus kinetic energy (TEDIV), both including enthalpy, geopotential, kinetic energy, and latent heat. Note that water in all states (gaseous *g*, liquid *l*, solid *s*) is considered, as well as latent heat of vapor and snow/ice. The acronyms that are not explicitly mentioned have their standard meaning, and a list of used symbols and acronyms is provided in Table 2.

**Table 2:** List of acronyms

AE	Atmospheric total energy
*C*	Celsius
*c*_a_	Isobaric specific heat capacity of dry air
*c*_l_	Specific heat capacity of liquid water
${\dot{C}}_{{\text{vl}}}$	Condensation rate from vapor to liquid cloud particles
${\dot{C}}_{{\text{vi}}}$	Condensation rate from vapor to ice cloud particles
${\dot{C}}_{{\text{li}}}$	Freezing rate from liquid to ice cloud particles
DSEDIV	Divergence of dry static plus kinetic energy transport
*E*	Evaporation rate at the surface (positive downward)
*F*_e_	Surface enthalpy flux associated with evaporation
*F*_S_	Net surface energy flux (latent plus sensible heat flux plus net radiation plus energetic effect of snowfall)
*g*	Gravitational acceleration
*h*_00_	Reference enthalpy of water
*K*	Kelvin
*k*	Atmospheric kinetic energy
*k*_s_	Atmospheric kinetic energy at the surface
LH	Latent heat flux
*L*_f_	Latent heat of fusion
*L*_s_	Latent heat of sublimation
*L*_v_	Latent heat of vaporization
*P*	Total precipitation (sum of rain and snow; positive downward)
*P*_rain_	Rain rate
*P*_snow_	Snowfall rate
*p*	Atmospheric pressure
${\dot{P}}_{{\text{vr}}}$	Column-integrated net conversion rate from vapor to rain
${\dot{P}}_{{\text{vs}}}$	Column-integrated net conversion rate from vapor to snow
${\dot{P}}_{{\text{ri}}}$	Column-integrated net conversion rate from rain to ice
${\dot{P}}_{{\text{ls}}}$	Column-integrated net conversion rate from liquid particles to snow
${\dot{P}}_{{\text{rs}}}$	Column-integrated net conversion rate from rain to snow
*q*_*v*_	Specific vapor content
*q*_l_	Specific liquid water content
*q*_f_	Specific frozen water (snow, ice) content
Rad_S_	Net radiation at the surface
Rad_TOA_	Net radiation at the top-of-atmosphere
SH	Sensible heat flux
SST	Sea surface temperature
*T*	Temperature
*T*_a_	Atmospheric temperature
*T*_p_	Temperature of precipitation
*T*_S_	Skin temperature
*T*_00_	Reference temperature
TEDIV	Divergence of moist static plus kinetic energy transport
$\Phi$	Geopotential
$\Phi$_s_	Surface geopotential
$$\overset{\lower0.5em\hbox{$\smash{\scriptscriptstyle\rightharpoonup}$}}{{\mathbf{v}}}$$	Horizontal wind vector

Equation (3) is almost identical to Mayer et al. (2017; their Eq. 19) but has surface geopotential and kinetic energy flux associated with precipitation and evaporation included. This equation is also consistent with equation D5 in (Kato et al. 2021) except that their formulation additionally allows for differing velocities of dry air and water particles (which we do not account for; see Sect. 3.1).

Equation (3) differs from the formulation of Trenberth and Fasullo (2018) as it only requires the energetic state of *P* and *E* at the surface, while theirs requires the full vertical profile of temperature at which condensation and evaporation occurs. This difference arises from the fact that Trenberth and Fasullo (2018) only include dry air and water vapor but not liquid water in their energy budget equations, i.e., water leaves the column where it condensates rather than when it hits the surface as rain (as is the case in Eq. 1 of this paper). The approach in Trenberth and Fasullo (2018) complicates the evaluation, especially given that there currently do not exist any observational products providing the height where precipitation is formed.

A diagnostic complication of Eq. (3) is that all terms involving mass exchanges or variations (*P*, *E*, storage, and divergence term) depend on the chosen reference enthalpy of water (*h*_00_) and the chosen reference temperature (*T*_00_). For the reference state of water, we make the typical choice to be liquid water at 0 °C and set this state to have zero enthalpy. Any other choice is valid (see Lauritzen et al. 2022 for variants of the total energy equation with different choices for h_00_ that are all equivalent), but for our application this seems convenient and is widely used. The effect of reference temperature on the evaluated terms can readily be seen for *F*_P_ and *F*_E_. The terms will be excessively large when choosing *K* scale. The effect of *T*_00_ on the storage and divergence terms is similar. This is because, e.g., the divergence term can be decomposed into a gradient term (independent of *T*_00_) and a mass divergence term that scales with energy and thus depends on *T*_00_ (in short form, $\nabla \cdot vT=v\nabla T+T\nabla \cdot v$; see extensive discussion in Mayer et al. 2017), only if the budget is mass-consistent (i.e., *P*, *E*, and lateral divergence of mass balance each other), and in the steady state, *T*_00_ drops out cleanly. Mass consistency can be achieved by mass corrections discussed in Sect. 3.2.4. However, the single terms remain dependent on *T*_00_, which is most pronounced for terms representing pure mass exchanges, such as *F*_P_ and *F*_E_. The effect of real mass variations arising from non-steady conditions on the magnitude of the diagnosed terms through *T*_00_ cannot be avoided but minimized by choosing *C* scale instead of *K* scale. However, as will be discussed next, simplifications typically made in atmospheric models help to avoid these complications.

#### Simplifications

Atmospheric reanalyses (as most atmospheric models, see Lauritzen et al. 2022) make several assumptions and simplifications in their energy and mass budgets. All water species are assumed to have the same heat capacity, namely that of dry air, and latent heats are taken as constants with the values at *T*_00_ = 0 °C (see Sect. 3.3 for discussion of the introduced error). Hydrometeors can thus be advected but carry the same specific enthalpy as dry air. In addition, moist physics parameterizations in many models including the IFS and JMA model do not modify total mass, which in consequence leads to unphysical sources and sinks of dry air to balance moist mass changes associated with net condensation. Thus, precipitation in these models does not carry energy (the energy is “conserved” in the appearing dry air), and consistent with this, there is no exchange of sensible heat with the environment and no dissipation associated with falling precipitation. However, latent heat associated with falling snow is taken into account. With these assumptions, Eq. (3) simplifies considerably to

4 $$\begin{aligned} & {\text{Rad}}_{{{\text{TOA}}}} - {\text{Rad}}_{{\text{S}}} - {\text{LH}} - {\text{SH}} - L_{{\text{f}}} \left( {T_{00} } \right)P_{{{\text{snow}}}} \\ & \quad = \frac{\partial }{\partial t}\frac{1}{g}\mathop \smallint \limits_{0}^{{p_{{\text{s}}} }} \left( {c_{{\text{a}}} (T_{{\text{a}}} - T_{00} ) + L_{{\text{v}}} \left( {T_{00} } \right)q_{{\text{v}}} + L_{{\text{f}}} \left( {T_{00} } \right)q_{{\text{f}}} + \phi_{{\text{s}}} + k} \right)\overset{\lower0.5em\hbox{$\smash{\scriptscriptstyle\rightharpoonup}$}}{{\mathbf{v}}} {\text{d}}p \\ & \quad + \nabla \cdot \frac{1}{g}\mathop \smallint \limits_{0}^{{p_{{\text{s}}} }} \left( {c_{{\text{a}}} (T_{{\text{a}}} - T_{00} ) + L_{{\text{v}}} \left( {T_{00} } \right)q_{{\text{v}}} + L_{{\text{f}}} \left( {T_{00} } \right)q_{{\text{f}}} + \phi + k} \right)\overset{\lower0.5em\hbox{$\smash{\scriptscriptstyle\rightharpoonup}$}}{{\mathbf{v}}} {\text{d}}p \\ \end{aligned}$$

This equation is consistent with the total energy budget equation as it is used in the IFS (sum of Eqs. 12.38 and 12.40 in ECMWF 2021b) and the JMA model. MERRA-2 follows a very similar total energy conservation equation as Eq. (4), but uses virtual temperature instead of *T*_*a*_ (Bosilovich et al. 2016). We dropped *h*_00_ in Eq. (4) as it is assumed zero, but a dependence on *T*_00_ in storage and divergence terms remains. As discussed above and will be shown below, the impact of the choice of *T*_00_ becomes small when mass consistency is ensured. It is also important to note that Eq. (4) is valid when all terms are evaluated from the same model-based data product. If the budget is evaluated using a mix of different products where some terms are evaluated using observational products which do not make the same simplifications, this will inevitably introduce inconsistencies.

#### Energy Conservation in Models and Reanalyses

After introducing the conservation equations of the physical and dynamical models underlying reanalyses, it is of interest to assess how well those are satisfied. Moist physics parametrizations in the IFS conserve total mass and enthalpy with the exception of a globally small <  = 0.1 W/m^2^ enthalpy error due to the handling of the mixed phase in convection. Importantly, the semi-Lagrangian advection scheme is non-conserving and introduces, depending on horizontal resolution, a spurious moisture and enthalpy source. As a result, the ERA5 energy budget exhibits a global mean imbalance of − 2.4 W/m^2^ (based on 2001–2020 mean global averages of 12-hourly short-term forecasts of vertical fluxes and atmospheric tendencies, where the latter is a proxy for the energetic effect of analysis increments), where the largest contribution (− 2.1 W/m^2^) stems from the non-closure of the moisture budget. This value is higher than those provided for two resolutions of the IFS (50 and 100 km versus ~ 31 km of ERA5) in Roberts et al. (2018), which indicates dependence on model resolution and the version of the IFS (Cy43R1 versus Cy41R2 in ERA5).

For consistency with the ERA5 estimate, we estimate the degree of non-closure of the energy budget in MERRA-2 by combining global mean net TOA and surface energy fluxes and total energy increments. The 2001–2020 average is − 1.0 W/m^2^, which represents the balance between physical tendencies and the tendencies introduces by the data assimilation. However, we note that the energy budget in MERRA-2 has a feature writing terms that exactly balance (Bosilovich et al. 2016). This is accomplished through the use of the Analysis Increment Update data assimilation scheme (Bloom et al. 1996) which acts as a numerical tendency alongside the physical tendencies in the forecast model’s budget equations together with an “energy fixer” for numerical dissipation.

We assessed energy conservation of the JMA model by looking at an AMIP-type run (JRA55-AMIP; Kobayashi et al. 2015), where no diagnostic complication from analysis increments arises. Global average of TOA minus net surface fluxes (i.e., the left side of Eq. 4) is 0.4 W/m^2^ over 2001–2012, which can be considered the model imbalance when neglecting atmospheric storage. This value is consistent with tests using JRA55 (taking account of the increments). We note that no corrections to improve budget closure have been applied during the production of JRA55-AMIP.

#### Mass Adjustment

As pointed out in numerous studies (e.g., Savijärvi 1982; Trenberth 1991, 1997; Chiodo and Haimberger 2010), analyzed winds from atmospheric (re)analyses do not satisfy the continuity equation discussed in Sect. 3.1, which has detrimental effects on the diagnosed energy budgets (Fig. 2 will illustrate this). The standard approach is a barotropic mass correction that is applied to the wind field at each time step TEDIV is computed and enforces satisfaction of the moist atmosphere’s mass budget (Eq. (1), see, e.g., Trenberth 1991). It is important to stress that the mass correction is applied to make the employed data self-consistent and not to adjust the mass budget towards some best estimate, e.g., of precipitation. Mayer et al. (2017) have laid out that application of this correction (and thus implicit inclusion of lateral energy fluxes associated with moisture) requires quantification of enthalpy fluxes associated with *P* and *E*. They also showed that neglect of those fluxes in conjunction with the typical choice for *K* temperature scale introduces a bias to the diagnosed fields on the order of 20–30 W/m^2^.

However, this approach for mass adjustment contrasts with the implementation of the mass budget in many models including the IFS, where lateral moisture transports do not accomplish a net energy transport except for that of latent heat, and, similarly, *P* and *E* do not carry any enthalpy except for latent heat (see Sect. 3.1). From that perspective, it is more in line with the underlying models to adjust the winds to satisfy Eq. (2), as done, e.g., by Chiodo and Haimberger (2010) who neglected *P* and *E* during their mass adjustment. We argue that this discrepancy can be reconciled by use of the simplified diagnostic equations proposed by Mayer et al. (2017) which consistently remove moisture enthalpies from lateral and vertical fluxes. This simplification assumes that moisture does not change temperature on its passage through the column and as a result moisture transports do not have a net energetic effect on the column (again, except for the release of latent heat). Hence, it is approximately equivalent to employ energy budget Eq. (4) in conjunction with winds adjusted to satisfy Eq. (2) (i.e., winds balance only surface pressure changes) or a variant of Eq. (4) with moisture enthalpy removed [similar to Eq. (24) in Mayer et al. (2017)] but with winds adjusted to satisfy Eq. (1) (i.e., winds additionally balance *P* + *E*) of this paper. Indeed, internal consistency of the ERA5 energy budget, i.e., satisfaction of Eq. (4) when computing all terms from ERA5 (not shown), is actually slightly better when using the latter variant. Hence, we will use that approach for ERA5 and JRA55 data for evaluations of TEDIV in the present paper as well. The ERA5-based TEDIV fields shown in this paper are thus the same as available from the Copernicus Data Store (CDS 2021) except that the latter do not account for atmospheric snow and ice.

Given that the MERRA-2 archive does not provide sub-monthly native grid 3D fields (see Sect. 2) needed for the mass adjustment discussed above, we resorted to a simplified mass correction that can be applied to vertically integrated divergences as described in Chiodo and Haimberger (2010). This method only takes into account the effect of the corrected mass flux divergence on TEDIV, but not the effect of modified advection on TEDIV. The latter effect is however small.

#### Evaluation

**Fig. 1:**
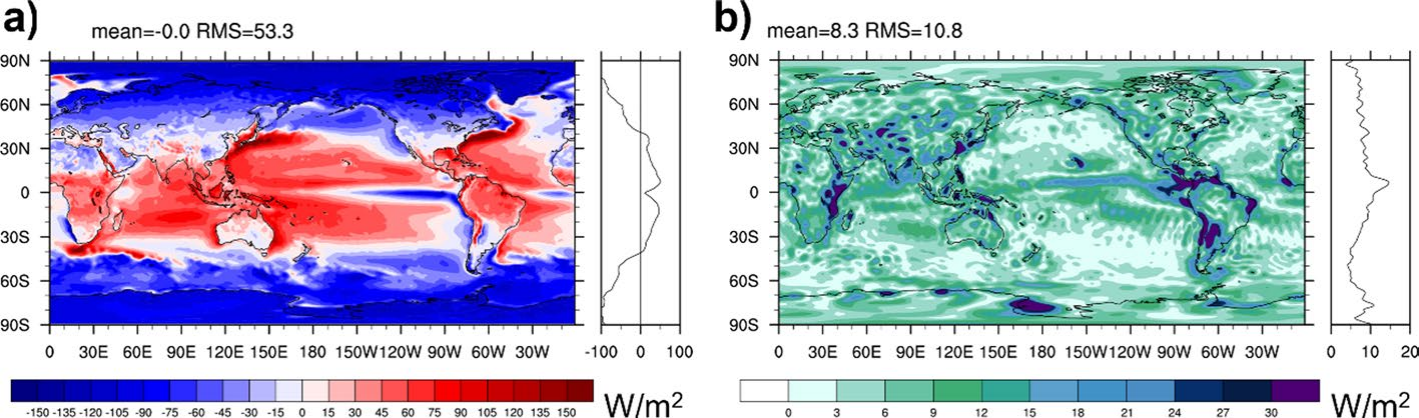
**a** Divergence of atmospheric moist static plus kinetic energy transports based on ERA5 averaged over 2001–2020 (truncated at T179); **b** spread in 2001–2020 mean TEDIV from ERA5, MERRA-2, and JRA55. Field in panel **b** is truncated at T63 to emphasize the larger scales

Figure 1a shows 2001–2020 average TEDIV from ERA5 following Eq. (4). As discussed in Sect. 3.2.4, winds have been adjusted such that they satisfy Eq. (4), moisture enthalpy has been removed from the transports, and computations were performed in Celsius scale. Main areas of divergence are warm tropical oceans and equatorial land masses, whereas convergence occurs over the equatorial cold tongues, extra tropical continents, and generally at high latitudes. The field in Fig. 1a has been evaluated using numerical methods as used in the IFS (as described in Mayer et al. 2021), which helps to reduce spectral noise and allows to truncate at relatively high wave numbers (here T179), i.e., the field effectively retains features at 1 degree resolution, which is much improved compared to earlier evaluations based on reanalyses that are based on spectral models (e.g., Trenberth and Stepaniak 2003; Edwards 2007; Mayer et al. 2017). Figure 1b presents an estimate of structural uncertainty in the divergence term, as estimated from the standard deviations (at each grid point) across the long-term average fields diagnosed from ERA5, MERRA-2, and JRA55. Large spread is found in the Intertropical Convergence Zone (ITCZ), where systematic differences in the divergent circulation between reanalyses likely affect the divergent energy transport. Spread over extratropical oceans, including western boundary currents with their strong divergences, is remarkably small. Spread over land is relatively large (especially in the tropics), and this is related to biases in the reanalyses and spectral artifacts projecting on larger scales.

**Fig. 2:**
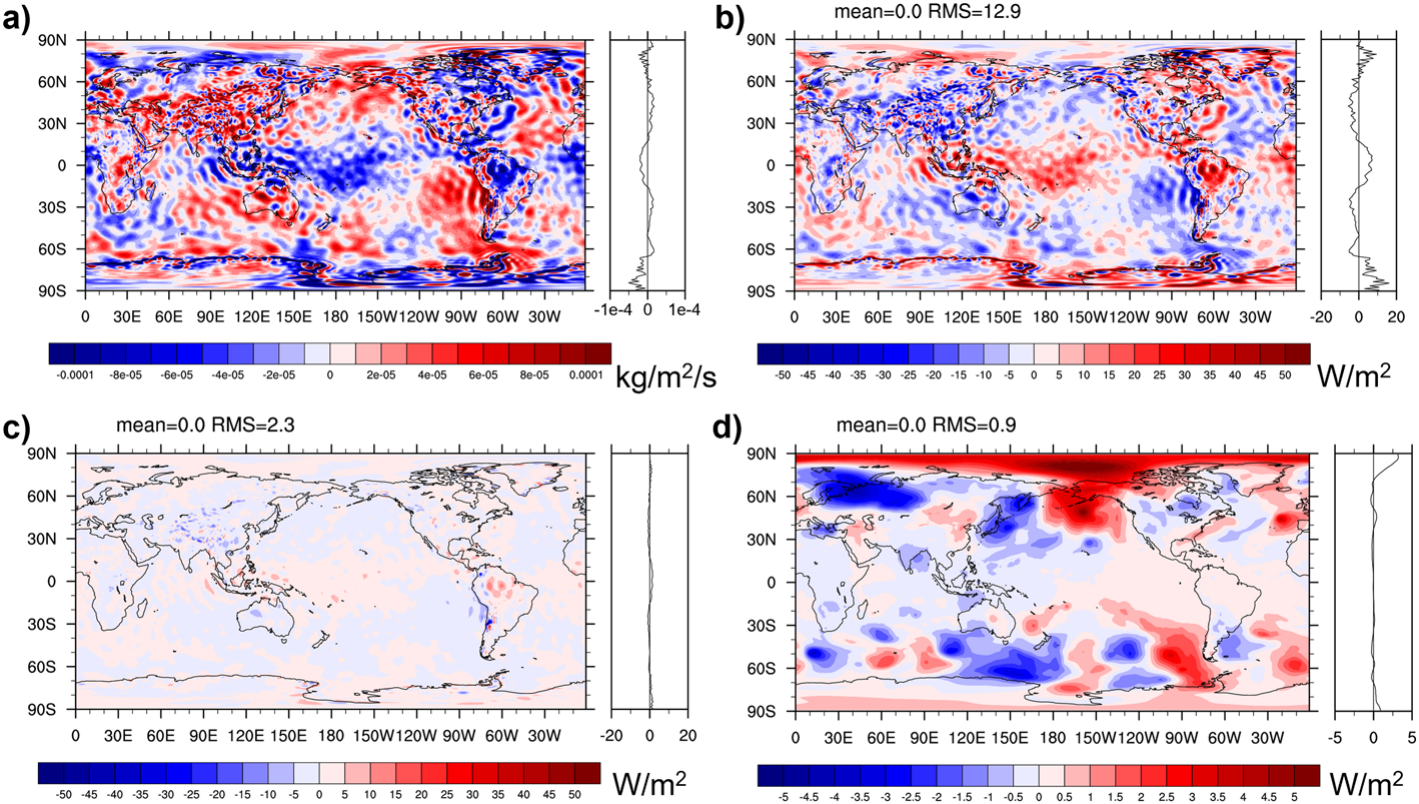
Impact of mass adjustment and different choices for reference temperature. **a** Vertically integrated mass flux divergence from ERA5 for 2015, **b** impact of mass-adjusting winds on TEDIV from ERA5 2015 (using Kelvin scale), **c** impact of mass-adjusting winds on TEDIV from ERA5 2015 (using Celsius scale), **d** difference of mass-adjusted TEDIV in 2015 using Kelvin or Celsius scale

The effect of mass adjustment and choice of *T*_00_ is illustrated in Fig. 2. Panel (a) shows the 2015 average mass flux divergence from ERA5. The shown pattern is unrealistic, as according to Eq. (2) the mass flux divergence should balance surface pressure variations, but the illustrated mass divergence patterns of order 10^–4^ kg/m^2^/s would imply annual surface pressure changes around 315 hPa/a. Panels (b) and (c) show the impact of a barotropic mass adjustment on TEDIV using either *K* or *C* temperature scale, respectively. The impact is larger in K scale, as expected from considerations in Sect. 3.2.1. The ambiguity of TEDIV arising from the choice of temperature scale becomes small after mass correction, as can be seen from panel (d). Differences only remain in the mid-latitudes, where real surface pressure variations within one year are non-negligible.

**Fig. 3:**
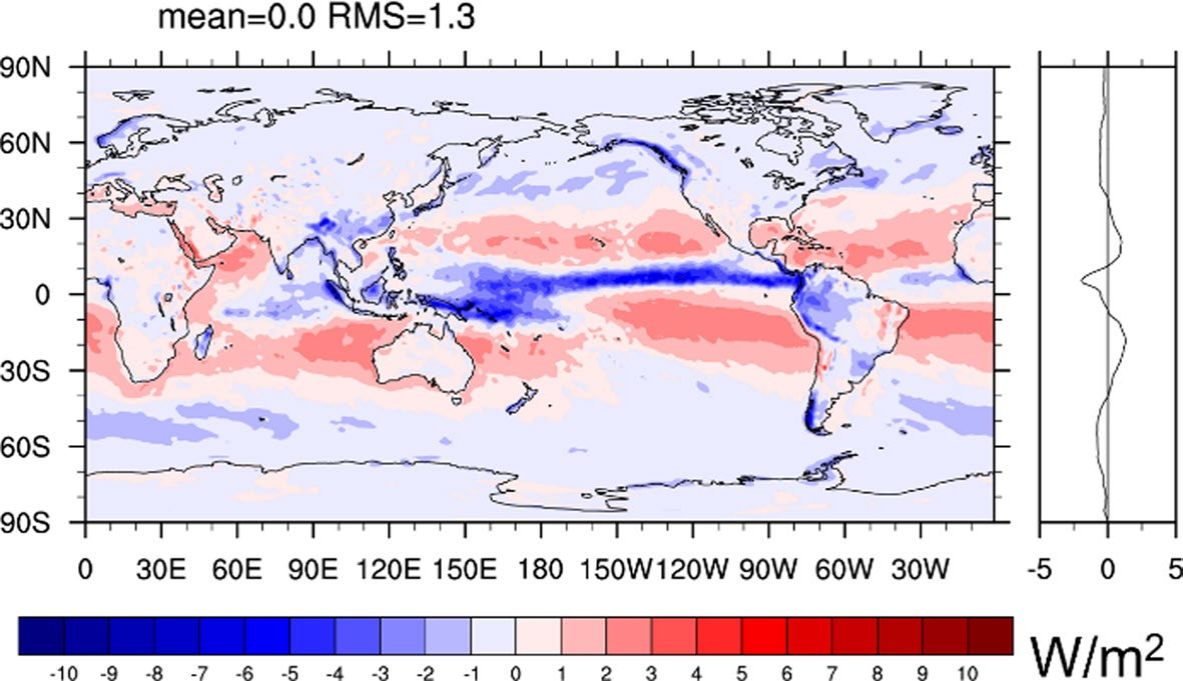
Difference of TEDIV computed following Mayer et al. (2017), i.e., with winds adjusted to satisfy Eq. (1) but moisture enthalpy taken out from Eq. (4), or following the IFS formulation, i.e., with winds adjusted to satisfy Eq. (2) and strictly following Eq. (4). Based on ERA5 data for 2015

Figure 3 shows the 2015 average difference in the energy flux divergence from ERA5 with the mass adjustment applied to satisfy Eq. (2) or to satisfy Eq. (1) but with 3D moisture enthalpy fluxes removed consistently (as proposed by Mayer et al. 2017). As expected from the discussion in 3.2.4, the difference is small, with a low-amplitude P–E pattern, which likely arises from the implicit consideration of potential energy of moisture (and its divergence) in the latter formulation (which is not taken into account in the IFS because of the discussed replacement of moisture with dry air). Results are thus very similar, if the formulations are self-consistent and mass consistency is ensured.

**Fig. 4:**
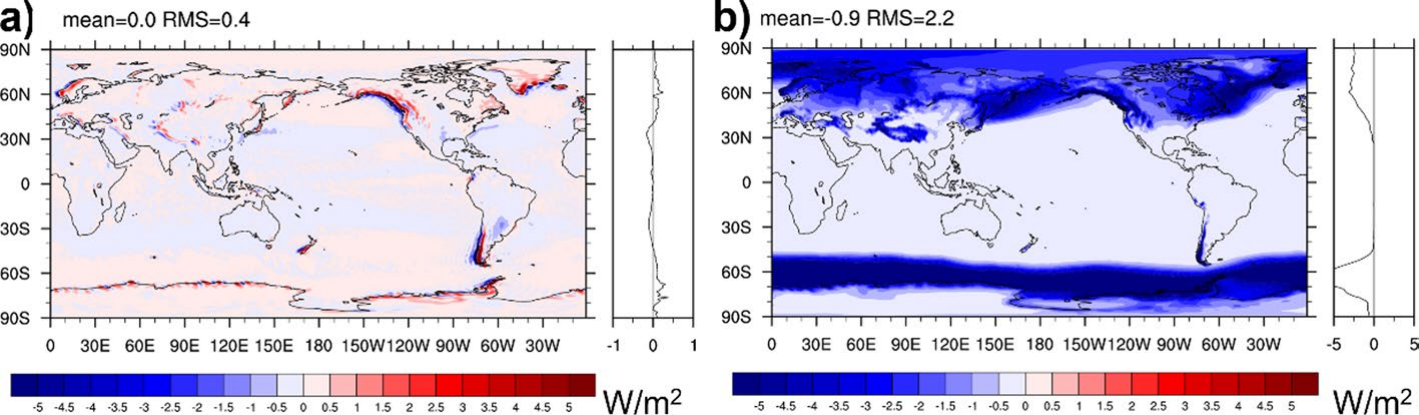
**a** Divergence of snow and ice-related latent heat transports, **b** ${L}_{{\text{f}}}{(T}_{{\text{00}}}{)P}_{{\text{snow}}}$ averaged over 2001–2020 based on ERA5 data

Figure 4 presents two fields that have often been neglected but should be included in evaluations of the total energy budget according to Eq. (4). Panel 4a shows the divergence of latent heat transports associated with snow and ice. Values are generally small except where the average atmospheric flow crosses mountain ranges, with convergence (divergence) on the windward (lee) side due to uplift and descent, respectively. Note the reverted sign compared to the divergence of atmospheric ice transport because the latent heat of snow and ice is relative to liquid water, and hence the contribution of *L*_f_ is negative. Panel 4b shows latent heat flux associated with snowfall, which represents a cooling of the surface [either by cooling the ocean or by depositing snow (because *L*_f_ < 0) on the land surface]. The global average effect is − 0.9 W/m^2^. As noted by Mayer et al. (2017), the typically considered surface fluxes Rad_S_ + SH + LH must be increased by this value to balance observed ocean warming.

**Fig. 5:**
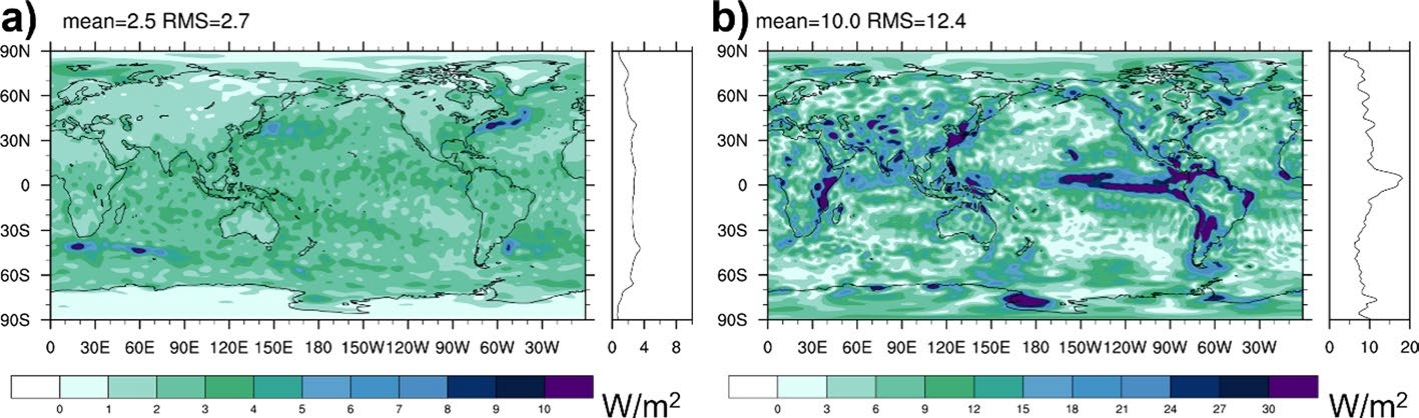
**a** Spread of TEDIV from the 10-member ERA5 ensemble during 2015, computed as spread of the 10 annual means; **b** inter-product spread in 2015 mean TEDIV from ERA5, MERRA-2, and JRA55

Direct validation of diagnosed atmospheric energy transports is impossible because no in situ observations of this quantity exist. The inter-product spread (Fig. 1b) is one indicator of uncertainty, but this could overestimate uncertainty if one product is an outlier but could also underestimate uncertainty in the case of similar structural biases. Since ERA5 is an ensemble product, we can also assess internal uncertainty from the ensemble spread, which is shown for the year 2015 in Fig. 5a. For a fair comparison, Fig. 5b shows the inter-product spread as presented in Fig. 1b but only for 2015. The magnitude of the ensemble spread of TEDIV from ERA5 is generally lower compared to the inter-product spread and there are regional differences, e.g., in the mid-latitudes. The latter is likely related to relatively high uncertainties in ERA5 associated with synoptic-scale activity which still stand out in a one-year average. The generally lower magnitude of the ERA5 spread can be explained by the largely random nature of the perturbations used for ensemble generation (Hersbach et al. 2020), a large fraction of which is averaged out when considering one-year means (the spread is considerably larger on monthly timescales; not shown). Figure 5b is very similar to Fig. 1b, with modestly enhanced magnitude (RMS increased by ~ 15%), which indicates that most of the inter-model spread on annual timescales arises from systematic differences and has relatively small temporal variability.

Another option for validation is to compare convergence of atmospheric energy over land to observed net TOA radiation, as there these quantities should balance locally in the long term if land heat uptake is neglected. Similarly, TOA radiation and atmospheric divergence can be combined to yield net surface energy flux, which in the long-term mean should be small everywhere over land. This validation approach will be adopted in Sect. 4, where the net surface energy flux will be discussed.

### Atmospheric Dry Static Energy Budget

The dry static energy (DSE) budget equation integrated over the atmosphere expresses the energy balance of the atmospheric column after water mass multiplied by latent heat has been subtracted from the reference state from Eq. (4) (Kato et al. 2021). The difference of the DSE equation from the total energy equation discussed in Sect. 3.2 is that water contribution appears as a diabatic heating term because lateral divergence and convergence of water mass is balanced by condensation and (re-)evaporation. In earlier studies, the DSE equation has been used to assess regions with a large energy balance residual when satellite derived data products are combined with reanalysis data (e.g., Kato et al. 2016, 2021).

To highlight fluxes that are not included in energy flux data products but needed for the energy balance in this section, we set up the DSE equation with assumptions that are mentioned in Sect. 3.2.2. In addition, because we assume that velocities of all water species are the same as the dry air velocity, the assumption yields a vertically integrated dry static energy equation which is simplified compared to Kato et al. (2021)

5 $$\frac{1}{g}\frac{\partial }{\partial t}\mathop \smallint \limits_{0}^{{p_{s} }} \left[ {\overline{{c_{{\text{a}}} }} T + \Phi_{{\text{s}}} + k} \right]{\text{d}}p + \frac{1}{g}\nabla \cdot \mathop \smallint \limits_{0}^{{p_{s} }} \overset{\lower0.5em\hbox{$\smash{\scriptscriptstyle\rightharpoonup}$}}{{\mathbf{v}}} \left[ {\overline{{c_{{\text{a}}} }} T + \Phi + k} \right]{\text{d}}p = \left( {{\text{Rad}}_{{{\text{TOA}}}} - {\text{Rad}}_{{\text{s}}} } \right) + L_{{\text{v}}} \left( {\dot{C}_{{{\text{vl}}}} + \dot{P}_{{{\text{vr}}}} } \right) + L_{{\text{s}}} \left( {\dot{C}_{{{\text{vi}}}} + \dot{P}_{{{\text{vs}}}} } \right) + L_{{\text{f}}} \left( {\dot{C}_{{{\text{li}}}} + \dot{P}_{{{\text{ri}}}} + \dot{P}_{{{\text{ls}}}} + \dot{P}_{{{\text{rs}}}} } \right) + {\text{SH}} + F_{{\text{E}}} + F_{{\text{P}}}$$

where the sum of enthalpy (with $\overline{{{\text{c}} }_{{\text{a}}}}$ representing mass-weighted specific heat as a function of atmospheric moisture) and surface geopotential is the dry static energy. Since the two terms on the lhs are typically evaluated using reanalysis data, we follow the simplification made in most models and use heat capacity of dry air (see Sect. 3.2.2). ${L}_{{\text{v}}}$, ${L}_{{\text{s}}}$, and ${L}_{{\text{f}}}$ are, respectively, the enthalpy of vaporization, sublimation, and fusion and ${\dot{{\text{P}}}}_{{\text{vr}}}$ is the rate of vapor condensed to raindrops that precipitate in the column and ${\dot{{\text{C}}}}_{{\text{vl}}}$ is the rate of vapor condensed to form cloud droplets. Similarly, *P* and *C* in the diabatic heating term indicate, respectively, precipitating and non-precipitating (i.e., clouds) hydrometeors. Subscripts *v*, *l*, *i*, *r*, and *s* indicate, respectively, water vapor, liquid, ice, rain, and snow. In reality, latent heats depend on temperature, but most models use constant latent heats (see Sect. 3.2.2). Kato et al. (2021) estimate the bias in diabatic heating rate when a constant latent heat of vaporization at 0 °C is used. The bias averaged over a year can be <− 5 Wm^−2^ in the tropics (because there typically ${L}_{{\text{v}}}\left({T}_{{\text{a}}}\right)<{L}_{{\text{v}}}\left({T}_{00}\right)$) and > + 5 Wm^−2^ in mid-latitude and polar regions (because there typically ${L}_{{\text{v}}}\left({T}_{{\text{a}}}\right)>{L}_{{\text{v}}}\left({T}_{00}\right)$). Note that additional underestimation of diabatic heating in mid-latitude and polar regions arises if the enhancement of latent heat release associated with snowfall is neglected (${L}_{{\text{s}}}\left({T}_{00}\right)>{L}_{{\text{v}}}\left({T}_{00}\right))$ (see Fig. 4b). Since in this section we derive diabatic heating rates based on an observational precipitation product, we use latent heat of vaporization at 0 °C for conversion.

Some earlier studies assume that hydrometeors formed in the column are precipitated out in the same column. Therefore, they neglect ${\dot{C}}_{{\text{vl}}}$, ${\dot{C}}_{{\text{vi}}}$, and ${\dot{C}}_{{\text{li}}}$ in Eq. (5). We used MERRA-2 data to estimate the energetic effect of ${\dot{C}}_{{\text{vl}}}$ (liquid water tendency due to dynamics multiplied by ${L}_{{\text{v}}}$), and long-term mean values range in ± 2W/m^2^ regionally (not shown). The energetic effect of ${\dot{C}}_{{\text{vi}}}$ and ${\dot{C}}_{{\text{li}}}$ is difficult to estimate separately, as the freezing and sublimation rates are typically not output by reanalyses. However, Fig. 4a provides an approximate estimation of the combined effect of the two terms, and it is concluded to be sizeable only close to high orography. The uncertainty in these estimates is expected to be large especially at a regional scale because of little observational constraint. Nevertheless, the result indicates that diabatic heating due to cloud particles may not be negligible for some regions.

*F*_E_ and *F*_P_ are generally not included in energy fluxes flux data products. These fluxes are needed to balance energy for the atmospheric column but add complexities in balancing regional energy fluxes using dry static energy equations. Mayer et al. (2017; their Fig. 7) showed that most of *F*_E_ + *F*_P_ is balanced by lateral moisture enthalpy transports on the lhs of Eq. (5). Thus, removing those from the divergence (as we do here, see Sect. 3.2.4) is assumed to remove most of the *F*_E_ + *F*_P_ pattern from the inferred diabatic heating. However, the imprint of *F*_E_ and *F*_P_ may implicitly be included when evaluating some terms of Eq. (5) using observational products, which do not make the same simplifications as made in reanalyses (see discussion in Sect. 3.2.2). With these considerations in mind, we drop *F*_E_ and *F*_P_ to write the DSE budget as

6 $$\frac{1}{g}\frac{\partial }{\partial t}\mathop \smallint \limits_{0}^{{p_{s} }} \left[ {c_{{\text{a}}} T\left( {1 - q_{{\text{v}}} - q_{{\text{l}}} - q_{{\text{f}}}} \right) + \Phi_{{\text{s}}} + k} \right]{\text{d}}p + \frac{1}{g}\nabla \cdot \mathop \smallint \limits_{0}^{{p_{s} }} \overset{\lower0.5em\hbox{$\smash{\scriptscriptstyle\rightharpoonup}$}}{{\mathbf{v}}} \left[ {c_{{\text{a}}} T\left( {1 - q_{{\text{v}}} - q_{{\text{l}}} - q_{{\text{f}}}} \right) + \Phi + k} \right]{\text{d}}p - \left( {{\text{Rad}}_{{{\text{TOA}}}} - {\text{Rad}}_{{\text{s}}} } \right) - {\text{SH}} = L_{{\text{v}}} \left( {\dot{C}_{{{\text{vl}}}} + \dot{P}_{{{\text{vr}}}} } \right) + L_{{\text{s}}} \left( {\dot{C}_{{{\text{vi}}}} + \dot{P}_{{{\text{vs}}}} } \right) + L_{{\text{f}}} \left( {\dot{C}_{{{\text{li}}}} + \dot{P}_{{{\text{ri}}}} + \dot{P}_{{{\text{ls}}}} + \dot{P}_{{{\text{rs}}}} } \right)$$

**Fig. 6:**
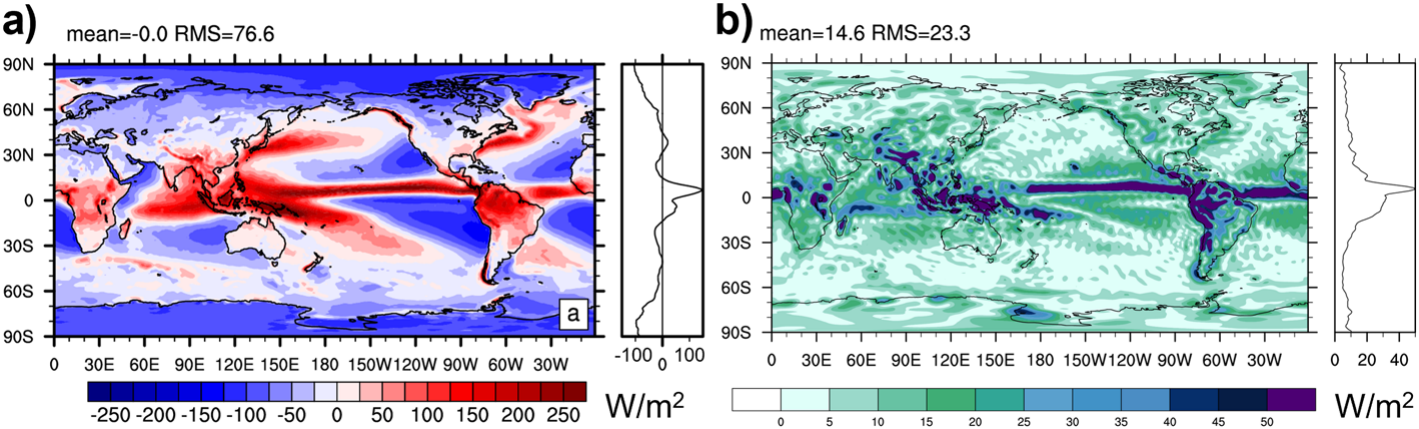
**a** Divergence of atmospheric dry static energy transports based on ERA5 averaged over 2001–2020 (truncated at T179); **b** spread in 2001–2020 mean DSEDIV from ERA5, MERRA-2, and JRA55. Field in panel **b** is truncated at T63 to emphasize the larger scales

Figure 6a shows the 2001–2020 mean divergence of DSE (DSEDIV) as written in Eq. (6) based on ERA5 data. Compared to the divergence of total energy transport (Fig. 1a), the field shows maxima where precipitation is maximal, i.e., in the ITCZ. Figure 6b shows the spread across the three DSE divergence estimates from ERA5, MERRA-2, and JRA55, as a measure of structural uncertainty. Uncertainties are generally larger compared to total energy (global RMS 23.3 W/m^2^ compared to 10.8 W/m^2^) and the spread peaks in the regions of deep convection in the tropics, which reflects the large uncertainty in DSEDIV. The spread of the total energy divergence is lower because total energy is conserved during latent heat release, i.e., in TEDIV, differences in DSE divergence are balanced by opposing differences in the divergence of latent heat transport (both terms are included in TEDIV) in the different products.

**Fig. 7:**
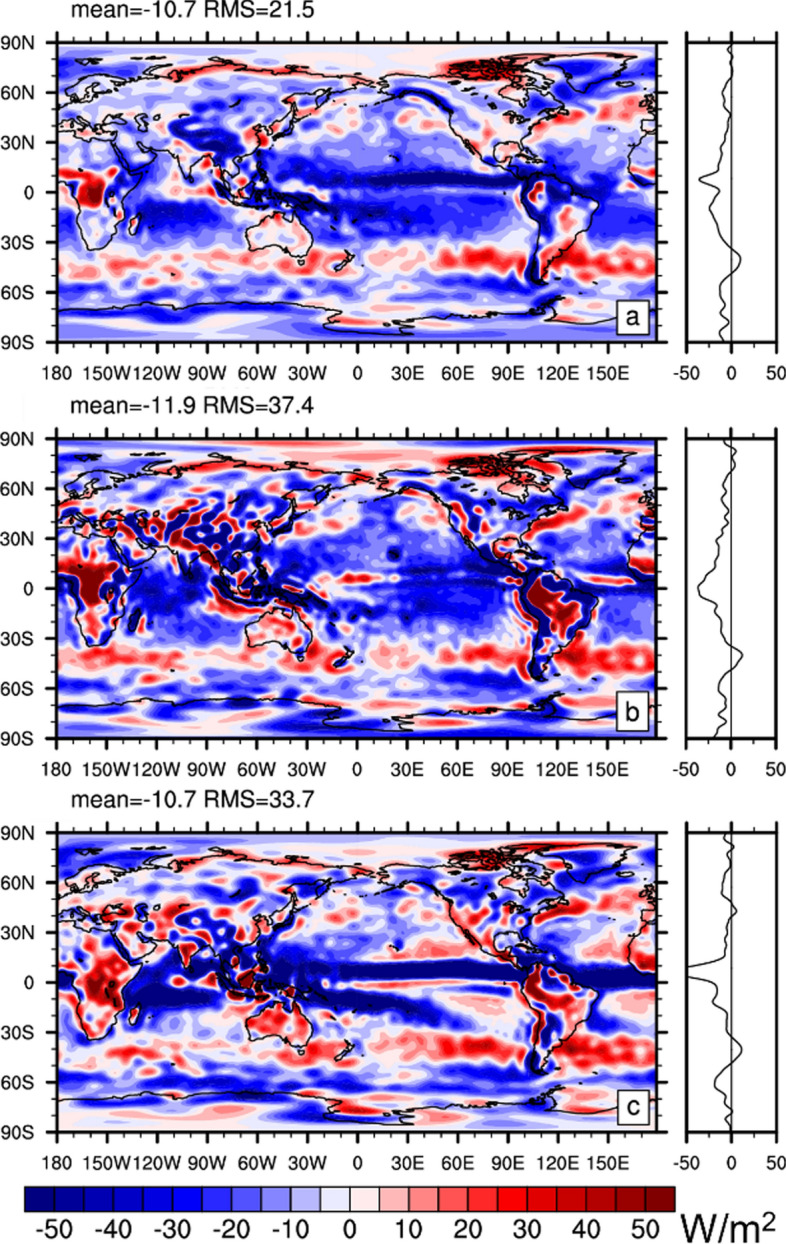
**a** Spatial distribution of atmospheric energy balance residual averaged from March 2000 through December 2018 computed with Eq. (6). Energy flux products used are Edition 4.2 EBAF (radiation), GPCP V3.2 (precipitation), SeaFlux (Sensible heat flux over ocean), ERA5 (sensible heat flux over land). **b** Same as (**a**) except that it uses MERRA-2 dry static and kinetic energy divergence and tendency and MERRA-2 sensible heat flux. **c** As (**a**) but uses JRA55 dry static and kinetic energy divergence and tendency. All fields are truncated at T63 for a fair comparison

Figure 7 shows the DSE budget residual (defined by bringing all terms of Eq. (6) to the rhs) averaged March 2000 through December 2018. Data products used here are CERES-EBAF (radiation), GPCP V3.2 (precipitation), SeaFlux v3 (Sensible heat flux over ocean), ERA5 (sensible heat flux over land), as well as ERA5, MERRA-2, and JRA55 (Dry static energy divergence and tendency). All fluxes in Eq. (6) are included. The DSE budget residual is considerable, with RMS values between 21.5 and 37.4 W/m^2^ (a considerable fraction of the RMS magnitude of the DSEDIV itself—compare to Fig. 6a), depending on the employed reanalysis product. All solutions for the residual exhibit a negative global mean, indicating inconsistencies between radiative fluxes, precipitation, and sensible heat flux data (note that global mean DSEDIV is 0 by construction and the DSE tendency is negligible). The residual patterns are not straightforward to interpret, but an imprint from tropical precipitation is clearly visible for all. This indicates that tropical DSEDIV from reanalyses is stronger than the diabatic heating suggested by precipitation from GPCP V3.2 and the radiative flux convergence from CERES-EBAF.

**Fig. 8:**
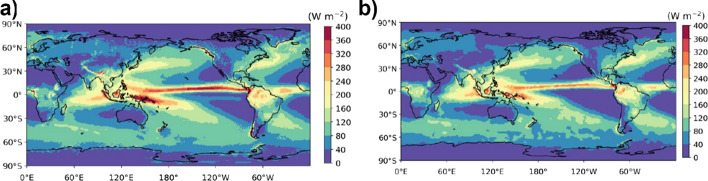
**a** Diabatic heating rate associated with water phase change (right side of Eq. 6) inferred from the sum of tendency and divergence terms, TOA and surface net irradiance, and sensible heat fluxes (i.e., left-hand side terms of Eq. 6) averaged from January 2001 through December 2018. **b** diabatic heating computed with GPCP V 3.2 precipitation for ${L}_{{\text{v}}}{\dot{P}}_{{\text{vr}}}+{L}_{{\text{s}}}{\dot{P}}_{{\text{vs}}}$ (i.e., the right-hand side of Eq. 6)

While the atmospheric energy balance residual shown in Fig. 7 remains, we can compute diabatic heating rate due to water phase changes by evaluating the lhs of Eq. (6) using a combination of reanalyses and observational products. Figure 8 (left) shows the diabatic heating rate derived from the left side of Eq. (6) using ERA5 reanalysis data (tendency and divergence terms), CERES-EBAF (TOA and surface irradiance), as well as Seaflux v3 and ERA5 sensible heat fluxes over ocean and land, respectively. The equivalent diabatic heating by precipitation derived from the right side of Eq. (6) is shown in the right panel of Fig. 8. In computing the right side of Eq. (6), the diabatic heating by non-precipitating hydrometeors and fusion terms are ignored. The diabatic heating rate estimated from the DSE budget and from GPCP averaged over different regions are shown in Table 3. Diabatic heating rates estimated from the DSE budget are generally higher than those based on GPCP (~ 14% in the global ocean mean and ~ 21% in the tropics). This is a substantial difference, still larger than can be expected through potential increase in GPCP guided by merging the currently best precipitation sensors over the oceans and considering the latest version of the precipitation products (Behrangi and Song 2020; Behrangi et al. 2023) and suggests a positive bias of the DSE-based estimates of diabatic heating.

**Table 3:** Global and regional mean diabatic heating rates averaged over the time period from January 2001 through December 2018

	Evaluate lhs of Eq. (6)		Evaluate rhs of Eq. (6)	
	Data product			
$$\frac{1}{g}\nabla \cdot {\int }_{0}^{{p}_{s}}\overset{\lower0.5em\hbox{$\smash{\scriptscriptstyle\rightharpoonup}$}}{{\mathbf{v}}}\left[{c}_{a}T+\Phi +k\right]{\text{d}}p$$	ERA5	MERRA-2		
${{\text{Rad}}}_{{\text{TOA}}}-{{\text{Rad}}}_{s}$	EBAF Ed4.2	EBAF Ed4.2		
${\text{SH}}$	SeaFlux 3.0 (Ocean), ERA5 (Land)	MERRA-2		
$L_{{\text{v}}} \left( {\dot{C}_{{{\text{vl}}}} + \dot{P}_{{{\text{vr}}}} } \right) +$ $L_{{\text{s}}} \left( {\dot{C}_{{{\text{vi}}}} + \dot{P}_{{{\text{vs}}}} } \right) +$ $L_{{\text{f}}} \left( {\dot{C}_{{{\text{li}}}} + \dot{P}_{{{\text{ri}}}} + \dot{P}_{{{\text{ls}}}} + \dot{P}_{{{\text{rs}}}} } \right)$			GPCP + MERRA-2	GPCP + ERA5
Global (Wm^−2^)	93.3	91.5	81.7	81.6
Ocean (Wm^−2^)	105.2	101.4	92.2	92.0
Land (Wm^−2^)	72.6	74.1	63.4	63.3
Northern hemisphere (Wm^−2^)	96.0	94.5	84.4	83.3
Southern hemisphere (Wm^−2^)	90.7	88.5	80.0	79.9
30°N–30°S (Wm^−2^)	110.4	108.3	91.2	91.0
30°N–60°N plus 30°S–60°S (Wm^−2^)	87.0	84.1	83.5	83.5
60°N–90°N plus 60°S–90°S (Wm^−2^)	46.9	48.8	41.2	41.2

Because the diabatic heating is derived from the sum of terms on the left side of Eq. (6), biases in dry static energy divergence and tendency, atmospheric net irradiance, and sensible heat flux affect the diabatic heating. Although the regional bias in each term is difficult to quantify, the comparison of diabatic heating derived from Eq. (6) and precipitation products can be used to infer the uncertainty in the estimate of the diabatic heating by precipitation. A thorough investigation is left for the future.

## Net Surface Energy Budget

The net surface energy flux (*F*_S_) is a key quantity, as it drives atmospheric circulation and energy transports, and similarly oceanic heat redistribution. Moreover, regional changes in *F*_S_ are a key contributor to regional energy imbalance of the climate system. Direct measurements of *F*_S_ via eddy covariance methods are scarce, but there exist several alternative approaches.

F_S_ can be diagnosed “directly” using satellite products of turbulent fluxes obtained via remotely sensed quantities input to bulk formulae (OAflux, J-OFURO, IFREMER, SeaFlux) and radiative fluxes (CERES-EBAF-sfc, CLARA-A3). Reanalyses output surface fluxes “directly” during short-term forecasts produced during data assimilation.

Alternatively, the total energy budget can be used to infer net surface energy flux *F*_S_ as a residual, i.e., by rearranging Eq. (4) and evaluating the lhs of the following equation:

7 $${\text{Rad}}_{{{\text{TOA}}}} - {\text{TEDIV}} - \frac{\partial }{\partial t}{\text{AE}} = {\text{Rad}}_{{\text{S}}} + {\text{LH}} + {\text{SH}} + L_{{\text{f}}} \left( {T_{00} } \right)P_{{{\text{snow}}}}$$

We define *F*_S_ as the sum of net surface radiation, turbulent surface fluxes, and the energetic effect of snowfall (i.e., the rhs of Eq. 7) The lhs of Eq. (7) is typically evaluated by combining net TOA radiation from a satellite product such as CERES-EBAF with atmospheric divergence and tendencies obtained from reanalyses. The advantage of this approach is that reanalysis-based analysis fields are strongly constrained by observations and thus are deemed more accurate than vertical fluxes output during the short-term forecasts. Another key feature of the residual approach is that, since global mean divergence vanishes and global mean $\frac{\partial }{\partial {\text{t}}}{\text{AE}}$ is very well constrained by observations (Johnson et al. 2023; Mayer et al. 2019; Von Schuckmann et al. 2022), the global mean bias of the inferred flux roughly equals that of the employed TOA flux product (i.e., < 1 W/m^2^).

**Fig. 9:**
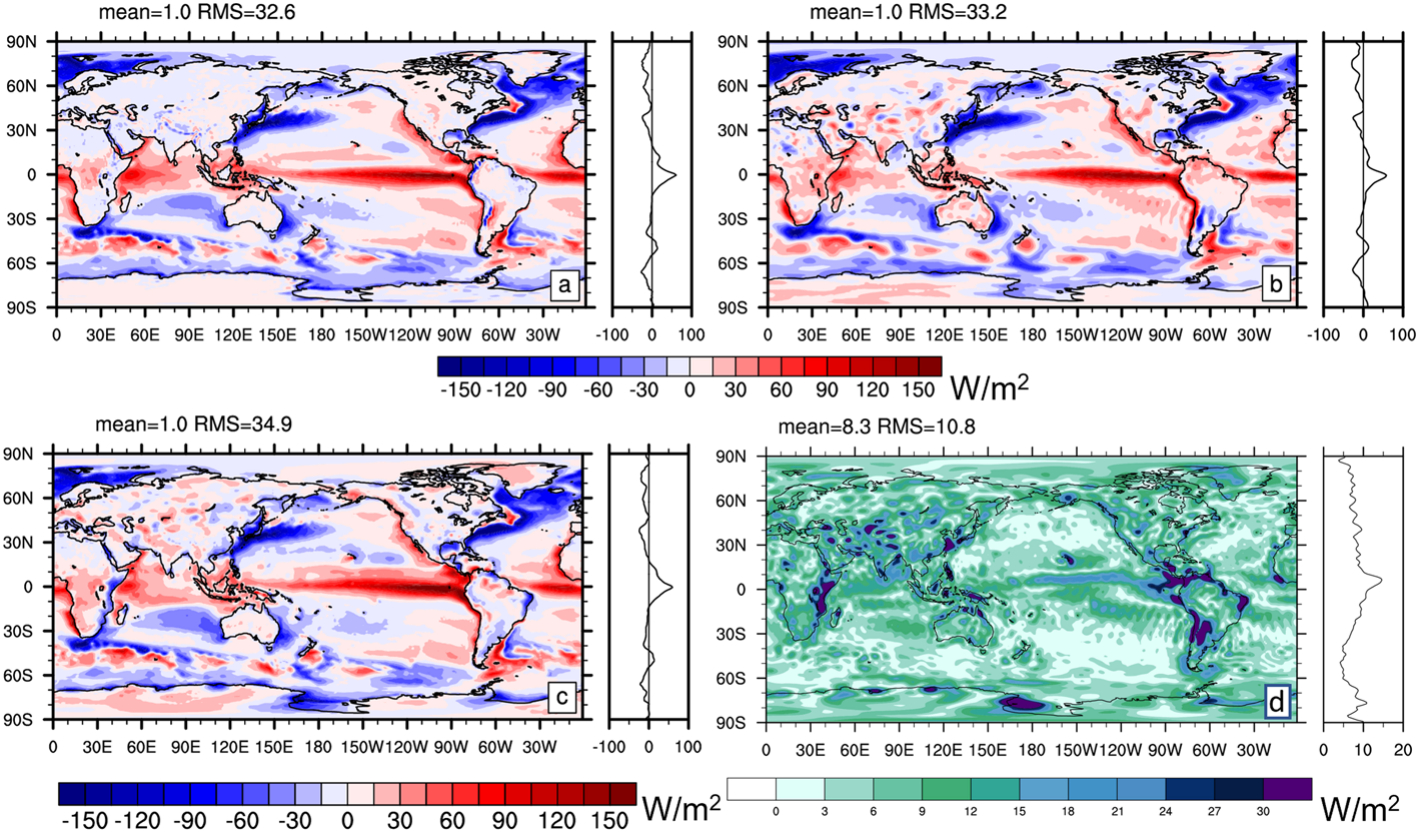
Inferred net surface energy flux over ocean based on CERES-EBAF-TOA and **a** ERA5 (truncated at T179), **b** JRA55 (truncated at T63), and **c** MERRA-2 (untruncated) data averaged over 2001–2020. Panel **d** at bottom right shows the spread (measured as standard deviation) across the three fields shown in (**a**)–(**c**)

We begin the evaluation with an inter-comparison of inferred *F*_S_ averaged 2001–2020 based on the atmospheric energy budgets from ERA5, MERRA-2, JRA55, as shown in Fig. 9a–c, respectively. The spread (Fig. 9d) is almost identical to that of the divergence (Fig. 1b), indicating that atmospheric storage and especially its inter-product spread is very small (not shown). *F*_S_ over land is expected to be small (< 1 Wm^−2^) locally on 20-year timescales, and hence patterns of inferred *F*_S_ over land inform about uncertainties. The spatial RMS of the long-term means over land ranges in ~ 13–19 W/m^2^ (Table 4), depending on the product. This represents an uncertainty estimate on the local scale, but we note that spectral noise is maximal over high topography as visible in fields based on ERA5 and JRA55 (both are based on spectral models) and thus contributes to uncertainty mostly over land. We thus we expect local errors over the ocean to be generally smaller. Comparison of Fig. 9a–c also reveals common errors such as the positive bias over central Africa present in all three estimates, which are not revealed by the inter-product spread. On a larger (continental) scale, the bias of inferred *F*_S_ becomes significantly smaller, ranging in ± 3 W/m^2^ except for the Maritime Continent, where all estimates have significant biases, likely due to the dominance of coastal areas with strong gradients (see Table 4). Global land and ocean averages of the three estimates exhibit fairly small biases, suggesting only small biases in global-scale ocean-to-land energy transport in all three reanalyses. The global mean *F*_S_ is 1 W/m^2^, as dictated by the global mean of CERES-EBAF-TOA fluxes. Note that the implied fluxes include the effect of snowfall (see rhs of Eq. 7). We can obtain the global average of surface net radiation plus turbulent heat fluxes (often used to approximate net surface energy flux) using the quantification of the snowfall term based on ERA5 data (global average is − 0.9 W/m^2^, see Fig. 4b), which is 1.9 W/m^2^ for 2001–2020.

**Table 4:** Satisfaction of physical constraints on a regional scale

Region	FS_inferred_ using CERES-EBAF-TOA combined with atmospheric budgets from		
	ERA5	MERRA-2	JRA55
South America	− 0.8	− 2.3	6.2
North America	2.4	− 2.2	3.0
Australia	2.7	0.8	12.4
Maritime Continent	− 8.8	20.8	32.7
Africa	1.4	5.1	9.3
Eurasia	− 3.1	0.9	3.1
Global land	− 0.6	1.1	5.4
Global Ocean	1.5	0.9	− 0.5
Global Ocean + Land	1.0	1.0	1.0
Global land spatial RMS value	13.2	18.8	19.0

Results are shown for inferred net surface energy flux [W/m^2^] combining CERES-EBAF-TOA fluxes and atmospheric budgets (divergence and tendency) from ERA5, MERRA-2, and JRA55, averaged over 2001–2020

**Fig. 10:**
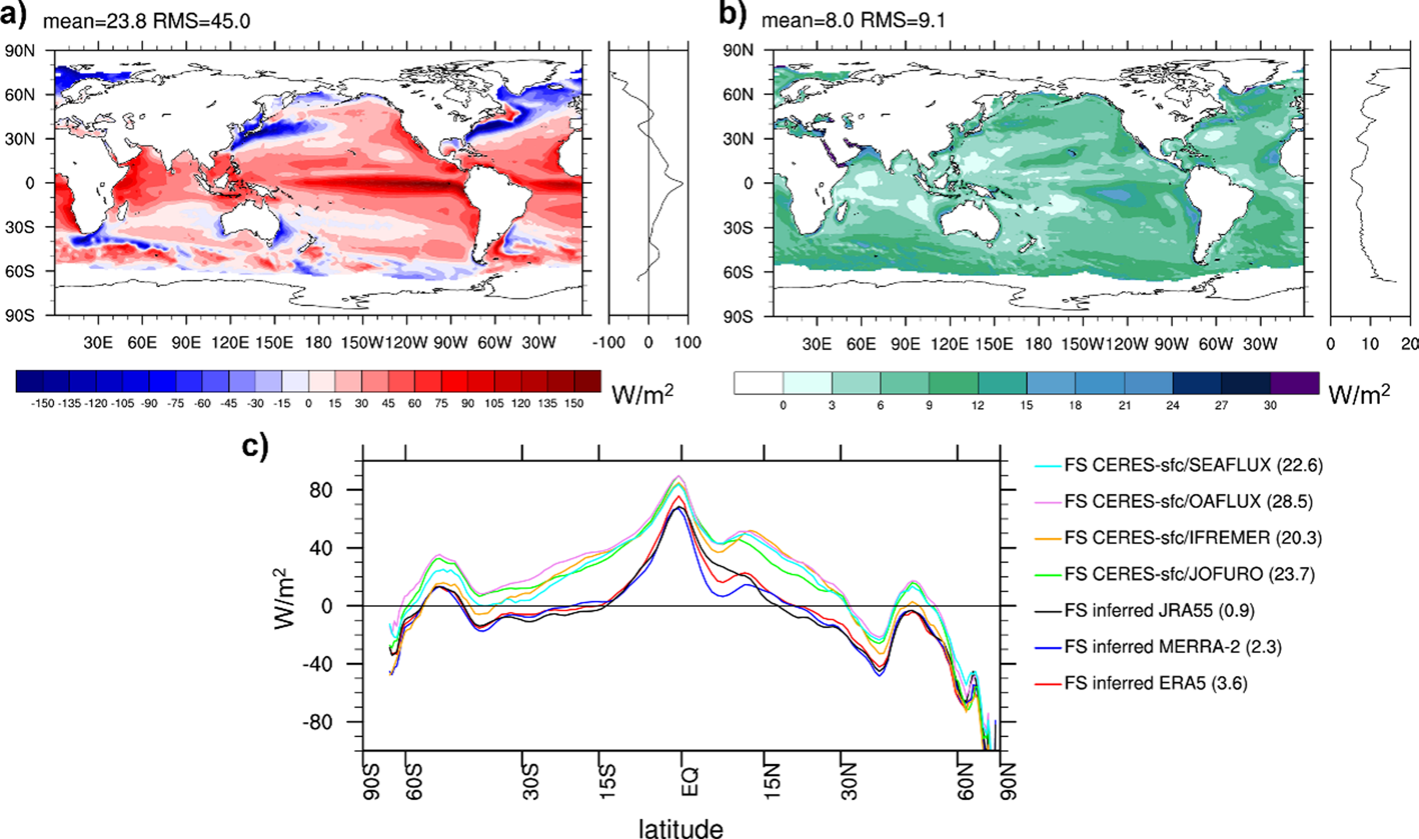
Net surface energy flux derived from satellite-based products (energetic effect of snowfall taken from ERA5); **a** ensemble mean of four estimates combining CERES-EBAF-sfc and SeaFlux, OAflux, IFREMER, J-OFURO, respectively; **b** ensemble spread (standard deviation) across the four estimates; **c** zonally averaged net surface energy flux over the ocean from satellite-based estimates and inferred flux estimates. Global averages over all grid points with valid data are provided in parentheses (units are W/m^2^). All estimates shown in (**c**) have the same spatiotemporal mask applied and are averaged over 2001–2017

Figure 10a presents the multi-product mean *F*_S_ obtained from four satellite products averaged over 2001–2017. For a clean comparison to the inferred fluxes, we added the energetic effect of snowfall based on ERA5 data (as shown in Fig. 4b) to the satellite-based radiative and turbulent fluxes. From comparison with Fig. 9a–c it is evident that the satellite-based estimates exhibit positive values (i.e., net ocean heat uptake) over much larger regions. Inter-product spread is relatively large over subtropical basins and the mid-latitudes and small in the Warm Pool regions (Fig. 10b). Figure 10c presents an inter-comparison of zonally averaged inferred and satellite-based F_S_ estimates. It reveals substantial differences between estimates from the two approaches, with the satellite-based fluxes being higher (i.e., more flux into the ocean) across almost all latitudes, with smaller differences in high latitudes. The satellite-based estimates exhibit strongly positive global mean values > 20 W/m^2^, which is a much higher value than can be expected from the removal of the grid points in the vicinity of sea ice with typically negative *F*_S_ values. This effect is estimated to be order 4–5 W/m^2^, as can be seen from a comparison of the quasi-global ocean means of inferred *F*_S_ in Fig. 10c to true ocean averages provided in Table 4. Thus, satellite-based F_S_ estimates exhibit a long-term global ocean mean bias order ~ 15 W/m^2^. Comparison of Rad_S_ from CERES-EBAF-sfc and CLARA-A3 shows very good agreement (global long-term mean difference − 2.2 W/m^2^ and spatial RMS of long-term mean difference is 5.3 W/m^2^) of the two products. It is noted that both products utilize similar approaches to estimate surface radiation, i.e., via radiative transfer simulations and the use of simultaneous AVHRR and CERES data for the estimation of broadband albedo in CLARA-A3. Apart from this, they are independent as they utilize different satellite input, auxiliary data and radiative transfer models. Thus, the agreement between both radiation records suggests that the main source of uncertainty are the turbulent fluxes.

Lastly, in Table 5 we present long-term global, land, and ocean mean *F*_s_ values from forecasts of different reanalysis products, which are listed in Table 1. Values range in − 11.2 to + 11.4 W/m^2^, with the largest values obtained from reanalyses with full data assimilation and relatively small values from the AMIP-type runs. All reanalyses except for NCEP R2 have the largest biases over ocean, where prescribed SSTs imply infinite heat capacity of the ocean and as a result lead to unrealistic fluxes. This indicates that assimilation of observational data into reanalyses arguably yields a more realistic atmospheric state but introduces discrepancies between observed and model climates that are reflected by unrealistically large vertical energy flux during the short-term forecasts.

**Table 5:** 2001–2010 average net surface energy flux in W/m^2^ obtained from reanalyses with varying amounts of data assimilated (based on short-term forecasts) and free AMIP-style runs of atmospheric models underlying some of the employed reanalyses (MERRA-2 AMIP and ERA20CM)

Region	ERA5	MERRA-2	JRA55	MERRA-2 AMIP	NCEP R2	20CRv3	ERA20C	ERA20CM	MERRA
Global land	1.0	0.8	0.5	1.1	8.1	1.2	0.8	0.9	1.7
Global Ocean	5.1	− 6.6	− 15.7	− 1.8	0.9	9.1	1.8	2.3	15.2
Global Ocean + Land	3.9	− 4.6	− 11.2	− 1.0	2.8	6.9	1.5	1.9	11.5

## Summary, Conclusions, and Challenges Ahead

This paper discussed frameworks to diagnose the atmospheric mass and energy budgets, including storage terms, vertical fluxes, and lateral transports. We detailed assumptions typically made in atmospheric models that underlie reanalysis products, which are essential data sets for some terms of the energy budget, most notably the lateral transports and their divergence. These assumptions simplify the diagnostic equations, and optimal use of the data can only be made if diagnostics take into account the models’ conservation equations in a consistent manner. For example, models typically use constant latent heats rather than more realistic temperature-dependent formulations, which should be then followed when using the data.

We evaluated the total and dry static energy budget using observational data and three atmospheric reanalyses ERA5, JRA55, and MERRA-2. Thereby, we illustrated the impact of mass corrections which are still needed despite the progress made in data quality over past decades. Specific attention has been paid to the sensitivity of results to the employed temperature scale, and it has been shown that the Celsius scale is preferable since it helps to minimize ambiguities that remain in diagnosed budgets after mass correction due to real mass variations.

All these assumptions and errors contribute to the regional energy balance residual, and a complication arises from the fact that different products, most notably reanalyses compared to observations, use different assumptions. As a consequence, when we compare individual estimates of the errors in different terms (e.g., Fig. 6) with the regional energy balance residual shown in Fig. 7, individual maps do not fully resemble the map of the regional energy balance residual. This means that errors are present in divergence, radiation, and turbulent flux products. While balancing regional energy using observation-based products is difficult, we believe two approaches are useful to move forward toward balancing regional energy budgets. One approach is, as demonstrated by L’Ecuyer et al. (2015) and similarly Mayer et al. (2019), to adjust each component of regional energy fluxes within their uncertainty. These studies demonstrate that regional energy fluxes can be balanced at a relatively large scale. Closing at smaller scales (e.g., 1° × 1°) is, however, difficult because of the increasing influence of energy and moisture transport in the local energy balance. Furthermore, estimating time- and regionally dependent errors for all products at a smaller temporal and spatial scales is difficult. Another challenge for the provision of realistic uncertainty estimates is that inter-product spread for the budget term in question can underestimate (due to common biases in different products) or overestimate (due to inferior quality of one or more employed products) true uncertainty. Nevertheless, the inter-product spread appears to provide a more realistic uncertainty estimate than the spread of current ensemble products such as ERA5, since ensemble perturbations do not take systematic errors (e.g., of the assimilating model) into account which seem to be dominant on the timescales considered here.

An alternative approach that we showed in this study is to use the energy balance equations to estimate one flux component from the sum of the rest of flux components as residual. While this approach guarantees to balance regional energy, all errors (arising from both shortcomings of the individual data sets but also inconsistencies arising from different assumptions made in different products) are in the residual. We showed the regional net surface energy flux estimated with net TOA flux and total energy divergence. Various metrics demonstrated a low bias of inferred *F*_S_ (across spatial scales, ranging from close to zero on global scale, over < 5 W/m^2^ on continental scale for ERA5 and MERRA-2 and ~ 15 W/m^2^ on the local scale of a 1 × 1 degree grid) compared to other approaches to estimate net surface energy flux, such as direct estimation from model output or satellite-based data. For the latter, the main source of uncertainty lies in the turbulent fluxes. Satellite-based estimates have been tuned to minimize differences with flux estimates from buoy-based measurements, whereby fluxes are obtained using bulk formulae. The large quasi-global biases of the satellite-based *F*_S_ estimates suggest that they are either overfitted to conditions at the buoy locations (which are largely in tropical seas) or the employed bulk formulae, which are known to have large uncertainties (Yu 2019), yield biased fluxes. Thus, in terms of long-term means inferred *F*_S_ fields appear to be of relatively high accuracy, and Mayer et al. (2022) have demonstrated that they are also superior in terms of temporal consistency. A major difficulty for evaluating surface fluxes is the limited availability of ground-truth data, such as measurements using eddy covariance methods over the ocean. As a consequence, we have to resort to the evaluation of physical constraints, which only exist for the long-term mean and only for the large scale over the ocean.

We also showed the regional diabatic heating associated with water estimated indirectly using radiative fluxes, dry static and kinetic energy divergence, and surface sensible heat flux. Both examples of inferring quantities as a residual take advantage of TOA flux constrained by ocean temperature measurements. The second example further extends the TOA flux constraint to surface radiative flux. Divergence terms in both examples use the same wind and temperature fields. The advantage of this residual approaches is that once assumptions and simplifications made in the employed data are accounted for in the diagnostic framework, inferred regional fluxes can be evaluated with measurements. For example, Trenberth and Fasullo (2017), Liu et al. (2020), or Mayer et al. (2022) used ocean heat transport measured at the RAPID section (Johns et al. 2011) and ocean temperature data in the North Atlantic to evaluate regional net surface fluxes derived as residual. The diabatic heating rate derived as residual can be evaluated by precipitation measurements. Results presented here indicate a positive bias of diabatic heating rates inferred from the dry static energy budget when compared to GPCP-based estimates, also when accounting for documented low biases of the latter based on the merger of the currently best and latest version of the precipitation products (Behrangi and Song 2020; Behrangi et al. 2023). However, the comparison in this paper is merely a demonstration of this application of the atmospheric energy budget and more work is needed to investigate the discrepancies found in more detail.

The evaluations presented here focused on long-term means. Assessing trends of inferred F_S_, an important application in the context of EEI research, still pushes the limits of the data, and only after ~ 2000 the involved products appear to be sufficiently stable (Loeb et al. 2022). Stable TOA fluxes such as those from CERES-EBAF are an essential ingredient for this, and their continuity into the future should have high priority. A remaining limiting factor of trend diagnostics is temporal stability of reanalyses, although progress has demonstrably been made over the years (e.g., Mayer et al. 2021). Nevertheless, for example TEDIV from ERA5 exhibits discontinuities in the late 1990s, which demonstrates the need for continued efforts in the areas of data rescue, homogenization, satellite data reprocessing activities, and bias estimation in future reanalyses, as advocated by Buizza et al. (2018).

It is expected that future reanalyses will increasingly adopt an Earth system approach, with atmosphere, ocean, sea ice, and land sub-systems being coupled. This will open new possibilities for coupled budget diagnostics, but the challenge of setting up a diagnostic framework that consistently (e.g., in terms of reference temperatures) tracks all relevant exchanges between the different compartments will remain. Generally, diagnostic frameworks need to take account of further development of data sets in the future and relaxation of simplification and assumptions in models. Evaluation efforts such as those presented here should be continued to document progress with updated data sets, and to inform about remaining challenges.

## References

[CR1] Bannon PR (2002) Theoretical foundations for models of moist convection. J Atmospheric Sci 59:1967–1982. 10.1175/1520-0469(2002)059%3c1967:TFFMOM%3e2.0.CO;2

[CR2] Behrangi A, Song Y (2020) A new estimate for oceanic precipitation amount and distribution using complementary precipitation observations from space and comparison with GPCP. Environ Res Lett 15:124042. 10.1088/1748-9326/abc6d1

[CR3] Behrangi A, Song Y, Huffmann GJ, Adler R (2023) Comparative analysis of the latest global oceanic precipitation estimates from GPM V07 and GPCP V3.2 products. J Hydrometeorol 25:293–309. 10.1175/JHM-D-23-0082.1

[CR4] Bentamy A, Grodsky SA, Katsaros K, Mestas-Nuñez AM, Blanke B, Desbiolles F (2013) Improvement in air–sea flux estimates derived from satellite observations. Int J Remote Sens 34:5243–5261. 10.1080/01431161.2013.787502

[CR5] Bloom S, Takacs L, Da Silva A, Ledvina D (1996) Data assimilation using incremental analysis updates. Mon Weather Rev 124:1256–1271. 10.1175/1520-0493(1996)124%3c1256:DAUIAU%3e2.0.CO;2

[CR6] Bosilovich MG, Lucchesi R, Suarez M (2016) MERRA-2: file specification. GMAO Off. Note No 9 Version 11, 73. http://gmao.gsfc.nasa.gov/pubs/office_notes

[CR7] Buizza R et al (2018) The EU-FP7 ERA-CLIM2 project contribution to advancing science and production of earth system climate reanalyses. Bull Am Meteorol Soc 99:1003–1014

[CR8] CDS (2021) Mass-consistent atmospheric energy and moisture budget monthly data from 1979 to present derived from ERA5 reanalysis. 10.24381/cds.c2451f6b

[CR9] Cheng L, Trenberth KE, Fasullo JT, Mayer M, Balmaseda M, Zhu J (2019) Evolution of ocean heat content related to ENSO. J Clim 32:3529–3556. 10.1175/JCLI-D-18-0607.1

[CR10] Chiodo G, Haimberger L (2010) Interannual changes in mass consistent energy budgets from ERA-Interim and satellite data. J Geophys Res. 10.1029/2009JD012049

[CR11] Collow ABM, Mahanama SP, Bosilovich MG, Koster RD, Schubert SD (2017) An evaluation of teleconnections over the United States in an ensemble of AMIP simulations with the MERRA-2 configuration of the GEOS atmospheric model. NASATM-2017-104606 47:78

[CR12] Curry JA et al (2004) Seaflux. Bull Am Meteorol Soc 85:409–424. 10.1175/BAMS-85-3-409

[CR13] ECMWF (2021a) IFS documentation CY47R3—part III Dynamics and numerical procedures. IFS Documentation CY47R3, IFS Documentation, ECMWF

[CR14] ECMWF (2021b) IFS documentation CY47R3—part IV Physical processes. IFS Documentation CY47R3, IFS Documentation, ECMWF

[CR15] Edwards JM (2007) Oceanic latent heat fluxes: consistency with the atmospheric hydrological and energy cycles and general circulation modeling. J Geophys Res Atmos. 10.1029/2006JD007324

[CR16] Gelaro R et al (2017) The modern-era retrospective analysis for research and applications, version 2 (MERRA-2). J Clim 30:5419–5454. 10.1175/JCLI-D-16-0758.1 10.1175/JCLI-D-16-0758.1 PMC6999672 32020988

[CR17] Global Modeling and Assimilation Office (GMAO) (2015a) MERRA-2 tavgM_2d_int_Nx: 2d, monthly mean, time-averaged, single-level, assimilation, vertically integrated diagnostics V5.12.4. 10.5067/FQPTQ4OJ22TL

[CR18] Global Modeling and Assimilation Office (GMAO) (2015b) MERRA-2 tavgM_2d_flx_Nx: 2d, monthly mean, time-averaged, single-level, assimilation, surface flux diagnostics V5.12.4. 10.5067/0JRLVL8YV2Y4

[CR19] Global Modeling and Assimilation Office (GMAO) (2015c) MERRA-2 tavgM_2d_rad_Nx: 2d, monthly mean, time-averaged, single-level, assimilation, radiation diagnostics V5.12.4. 10.5067/OU3HJDS973O0

[CR20] Gosnell R, Fairall CW, Webster PJ (1995) The sensible heat of rainfall in the tropical ocean. J Geophys Res Oceans 100:18437–18442. 10.1029/95JC01833

[CR21] Hersbach H, Peubey C, Simmons A, Berrisford P, Poli P, Dee D (2015) ERA-20CM: a twentieth-century atmospheric model ensemble. Q J R Meteorol Soc 141:2350–2375. 10.1002/qj.2528

[CR22] Hersbach H et al (2020) The ERA5 global reanalysis. Q J R Meteorol Soc 146:1999–2049. 10.1002/qj.3803

[CR23] Huffman GJ, Adler RF, Behrangi A, Bolvin DT, Nelkin EJ, Gu G, Ehsani MR (2023) The new version 3.2 global precipitation climatology project (GPCP) monthly and daily precipitation products. J Clim 36:7635–7655. 10.1175/JCLI-D-23-0123.1

[CR24] JMA (2007) Outline of the operational numerical weather prediction at the Japan Meteorological Agency. Appendix to WMO Technical Progress Report on the Global Data-Processing and Forecasting System and Numerical Weather Prediction. JMA

[CR25] Johns WE et al (2011) Continuous, array-based estimates of Atlantic Ocean heat transport at 26.5 N. J Clim 24:2429–2449. 10.1175/2010JCLI3997.1

[CR26] Johnson GC, Landerer FW, Loeb NG, Lyman JM, Mayer M, Swann AL, Zhang J (2023) Closure of earth’s global seasonal cycle of energy storage. Surv Geophys. 10.1007/s10712-023-09797-6 10.1007/s10712-023-09797-6 PMC11671433 39734428

[CR27] Kanamitsu M, Ebisuzaki W, Woollen J, Yang S-K, Hnilo JJ, Fiorino M, Potter GL (2002) NCEP–DOE AMIP-II reanalysis (R-2). Bull Am Meteorol Soc 83:1631–1644. 10.1175/BAMS-83-11-1631

[CR28] Karlsson K-G et al. (2023) CLARA-A3: the third edition of the AVHRR-based CM SAF climate data record on clouds, radiation and surface albedo covering the period 1979 to 2023. Earth Syst Sci Data. 10.5194/essd-15-4901-2023

[CR29] Kato S, Xu K-M, Wong T, Loeb NG, Rose FG, Trenberth KE, Thorsen TJ (2016) Investigation of the residual in column-integrated atmospheric energy balance using cloud objects. J Clim 29:7435–7452. 10.1175/JCLI-D-15-0782.1

[CR30] Kato S et al. (2018) Surface irradiances of edition 4.0 clouds and the earth’s radiant energy system (CERES) energy balanced and filled (EBAF) data product. J Clim 31:4501–4527. 10.1175/JCLI-D-17-0523.1

[CR31] Kato S, Loeb NG, Fasullo JT, Trenberth KE, Lauritzen PH, Rose FG, Rutan DA, Satoh M (2021) Regional energy and water budget of a precipitating atmosphere over ocean. J Clim 34:4189–4205. 10.1175/JCLI-D-20-0175.1

[CR32] Kobayashi S et al (2015) The JRA-55 reanalysis: general specifications and basic characteristics. J Meteorol Soc Jpn Ser II 93:5–48. 10.2151/jmsj.2015-001

[CR33] Kosaka Y et al (2024) The JRA-3Q reanalysis. J Meteorol Soc Jpn Ser II 102:49–109. 10.2151/jmsj.2024-004

[CR100] Lauritzen, PH et al (2018) NCAR release of CAM-SE in CESM2.0: A reformulation of the spectral element dynamical core in dry-mass vertical coordinates with comprehensive treatment of condensates and energy. J Adv Model Earth Syst 10:1537–1570. 10.1029/2017MS001257

[CR34] Lauritzen PH et al (2022) Reconciling and improving formulations for thermodynamics and conservation principles in Earth System Models (ESMs). J Adv Model Earth Syst 1(4):e2022MS003117

[CR35] L’Ecuyer TS et al (2015) The observed state of the energy budget in the early twenty-first century. J Clim 28:8319–8346. 10.1175/JCLI-D-14-00556.1

[CR36] Liu C et al (2020) Variability in the global energy budget and transports 1985–2017. Clim Dyn 55:3381–3396. 10.1007/s00382-020-05451-8

[CR37] Loeb NG et al (2018) Clouds and the earth’s radiant energy system (CERES) energy balanced and filled (EBAF) top-of-atmosphere (TOA) edition-4.0 data product. J Clim 31:895–918. 10.1175/JCLI-D-17-0208.1

[CR38] Loeb NG et al (2022) Evaluating twenty-year trends in Earth’s energy flows from observations and reanalyses. J Geophys Res Atmos 127:e2022JD036686. 10.1029/2022JD036686

[CR39] Malardel S, Diamantakis M, Agusti-Panareda A, Flemming J (2019) Dry mass versus total mass conservation in the IFS. ECMWF Technical Memorandum 849

[CR40] Mauder M, Foken T, Cuxart J (2020) Surface-energy-balance closure over land: a review. Bound-Layer Meteorol 177:395–426. 10.1007/s10546-020-00529-6

[CR41] Mayer J, Mayer M, Haimberger L (2021) Consistency and homogeneity of atmospheric energy, moisture, and mass budgets in ERA5. J Clim 34:3955–3974. 10.1175/JCLI-D-20-0676.1

[CR42] Mayer J, Mayer M, Haimberger L, Liu C (2022) Comparison of surface energy fluxes from global to local scale. J Clim 35:4551–4569. 10.1175/JCLI-D-21-0598.1

[CR43] Mayer M, Haimberger L, Edwards JM, Hyder P (2017) Toward consistent diagnostics of the coupled atmosphere and ocean energy budgets. J Clim 30:9225–9246. 10.1175/JCLI-D-17-0137.1

[CR44] Mayer M, Tietsche S, Haimberger L, Tsubouchi T, Mayer J, Zuo H (2019) An improved estimate of the coupled Arctic energy budget. J Clim 32:7915–7934. 10.1175/JCLI-D-19-0233.1

[CR45] Peixoto JP, Oort AH (1992) Physics of climate. American Institute of Physics Melville, New York, p 520

[CR46] Poli P et al (2016) ERA-20C: an atmospheric reanalysis of the twentieth century. J Clim 29:4083–4097. 10.1175/JCLI-D-15-0556.1

[CR47] Rienecker MM et al (2011) MERRA: NASA’s modern-era retrospective analysis for research and applications. J Clim 24:3624–3648. 10.1175/JCLI-D-11-00015.1

[CR48] Roberts CD, Senan R, Molteni F, Boussetta S, Mayer M, Keeley SP (2018) Climate model configurations of the ECMWF integrated forecasting system (ECMWF-IFS cycle 43r1) for HighResMIP. Geosci Model Dev 11:3681–3712. 10.5194/gmd-11-3681-2018

[CR49] Savijärvi H (1982) The mass balance in diagnostic studies: an example of analysed and forecast data calculations. Tellus 34:540–544. 10.3402/tellusa.v34i6.10839

[CR50] Slivinski LC et al (2019) Towards a more reliable historical reanalysis: Improvements for version 3 of the Twentieth Century Reanalysis system. Q J R Meteorol Soc 145:2876–2908

[CR51] Takacs LL, Suárez MJ, Todling R (2016) Maintaining atmospheric mass and water balance in reanalyses. Q J R Meteorol Soc 142:1565–1573. 10.1002/qj.2763 29643569 10.1002/qj.2763 PMC5890449

[CR52] Tomita H, Hihara T, Kako S, Kubota M, Kutsuwada K (2019) An introduction to J-OFURO3, a third-generation Japanese ocean flux data set using remote-sensing observations. J Oceanogr 75:171–194. 10.1007/s10872-018-0493-x

[CR53] Trenberth K (1991) Climate diagnosics from global analyses: conservation of mass in ECMWF analyses. J Clim 4:707–722. 10.1175/1520-0442(1991)004%3c0707:CDFGAC%3e2.0.CO;2

[CR54] Trenberth KE (1997) Using atmospheric budgets as a constraint on surface fluxes. J Clim 10:2796–2809. 10.1175/1520-0442(1997)010%3c2796:UABAAC%3e2.0.CO;2

[CR55] Trenberth KE, Stepaniak DP (2003) Seamless poleward atmospheric energy transports and implications for the hadley circulation. J Clim 16:3706–3722. 10.1175/1520-0442(2003)016%3c3706:SPAETA%3e2.0.CO;2

[CR56] Trenberth KE, Fasullo JT (2017) Atlantic meridional heat transports computed from balancing Earth’s energy locally. Geophys Res Lett 44:1919–1927. 10.1002/2016GL072475

[CR57] Trenberth KE, Fasullo JT (2018) Applications of an updated atmospheric energetics formulation. J Clim 31:6263–6279. 10.1175/JCLI-D-17-0838.1

[CR58] Trenberth KE, Zhang Y, Fasullo JT, Cheng L (2019) Observation-based estimates of global and basin ocean meridional heat transport time series. J Clim 32:4567–4583. 10.1175/JCLI-D-18-0872.1

[CR59] Von Schuckmann K et al (2022) Heat stored in the Earth system 1960–2020: where does the energy go? Earth Syst Sci Data 2022:1675–1709. 10.5194/essd-15-1675-2023

[CR60] Yu L (2019) Global air–sea fluxes of heat, fresh water, and momentum: energy budget closure and unanswered questions. Annu Rev Mar Sci 11:227–248. 10.1146/annurev-marine-010816-060704 10.1146/annurev-marine-010816-060704 30156969

[CR61] Yu L, Weller RA (2007) Objectively analyzed air–sea heat fluxes for the global ice-free oceans (1981–2005). Bull Am Meteorol Soc 88:527–540. 10.1175/BAMS-88-4-527

